# Context-Dependent Functional Divergence of the Notch Ligands DLL1 and DLL4 *In Vivo*

**DOI:** 10.1371/journal.pgen.1005328

**Published:** 2015-06-26

**Authors:** Kristina Preuße, Lena Tveriakhina, Karin Schuster-Gossler, Cláudia Gaspar, Alexandra Isabel Rosa, Domingos Henrique, Achim Gossler, Michael Stauber

**Affiliations:** 1 Institut für Molekularbiologie OE5250, Medizinische Hochschule Hannover, Hannover, Germany; 2 Instituto de Medicina Molecular, Faculdade de Medicina da Universidade de Lisboa, Lisboa, Portugal; The University of North Carolina at Chapel Hill, UNITED STATES

## Abstract

Notch signalling is a fundamental pathway that shapes the developing embryo and sustains adult tissues by direct communication between ligand and receptor molecules on adjacent cells. Among the ligands are two Delta paralogues, DLL1 and DLL4, that are conserved in mammals and share a similar structure and sequence. They activate the Notch receptor partly in overlapping expression domains where they fulfil redundant functions in some processes (e.g. maintenance of the crypt cell progenitor pool). In other processes, however, they appear to act differently (e.g. maintenance of foetal arterial identity) raising the questions of how similar DLL1 and DLL4 really are and which mechanism causes the apparent context-dependent divergence. By analysing mice that conditionally overexpress DLL1 or DLL4 from the same genomic locus (*Hprt*) and mice that express DLL4 instead of DLL1 from the endogenous *Dll1* locus (*Dll1^Dll4ki^*), we found functional differences that are tissue-specific: while DLL1 and DLL4 act redundantly during the maintenance of retinal progenitors, their function varies in the presomitic mesoderm (PSM) where somites form in a Notch-dependent process. In the anterior PSM, every cell expresses both Notch receptors and ligands, and DLL1 is the only activator of Notch while DLL4 is not endogenously expressed. Transgenic DLL4 cannot replace DLL1 during somitogenesis and in heterozygous *Dll1^Dll4ki/+^* mice, the *Dll1^Dll4ki^* allele causes a dominant segmentation phenotype. Testing several aspects of the complex Notch signalling system *in vitro*, we found that both ligands have a similar trans-activation potential but that only DLL4 is an efficient *cis*-inhibitor of Notch signalling, causing a reduced net activation of Notch. These differential *cis*-inhibitory properties are likely to contribute to the functional divergence of DLL1 and DLL4.

## Author Summary

Notch signalling relies on binding of a ligand to a Notch receptor, both residing on the surfaces of neighbouring cells. This interaction forwards a signal into the receptor-expressing cell, this way coordinating cells in many biological processes such as the segmentation of the axial skeleton. Mammals possess four Notch-activating ligands–including DLL1 and DLL4 -expressed in diverse, partially overlapping regions. Whether the different ligands trigger quantitatively or qualitatively distinct Notch responses is largely unknown. In order to directly compare both ligands we generated transgenic mice that express DLL1 or DLL4 in identical patterns. These mice uncover that only DLL1 but not DLL4 can mediate regular segmentation of the embryo. In experiments with cultured cells expressing either ligand and Notch, we found that the functional difference observed is unlikely to depend on differences in the activation of Notch. Rather, the unsuspected but strong difference between both ligands in *cis*-inhibition, i.e. repression of Notch by a ligand expressed in the same cell as the receptor, a process described in the fruitfly but not in mammals and not for DLL4 provides a possible explanation for the divergence in tissues that coexpress ligand and receptor.

## Introduction

The Notch signalling pathway mediates local interactions between adjacent cells and thereby regulates numerous developmental processes in a wide variety of different tissues throughout the animal kingdom [reviewed in 1–7]. The Notch gene of *Drosophila* and its vertebrate homologues encode large transmembrane proteins that act as receptors at the surface of the cell. They interact with transmembrane ligand proteins on the surface of neighbouring, signal-sending cells (i.e. in *trans*) encoded by the *Delta* and *Serrate* (called *Jagged* in vertebrates) genes. Upon ligand binding, the intracellular domain of Notch (NICD) is proteolytically released, translocates to the nucleus, interacts with the transcriptional regulator Suppressor of Hairless ([Su(H)]; CSL proteins in vertebrates) and activates the transcription of downstream target genes [8–14]. Ligands coexpressed with the Notch receptor in signal-receiving cells (i.e. in *cis*) are capable of interacting with Notch and attenuate the signal strength [15–17, reviewed in 18].

Vertebrates possess several Notch receptors and ligands. The mouse genome encodes four Notch (NOTCH1–4), three Delta (DLL1, DLL3 and DLL4) and two Jagged (JAG1 and JAG2) proteins. Among the DLL proteins, only DLL1 and DLL4 function as Notch-activating ligands [19–21]. As paralogues, DLL1 and DLL4 are similar in sequence (47% identical plus 14% similar amino acids), size and domain structure [22]. Both contain a DSL domain, which is essential for the interaction with Notch [23,24], as well as eight EGF-like repeats in their extracellular domain and have a short intracellular domain with a C-terminal PDZ binding motif. *Dll1* and *Dll4* are expressed both in discrete and overlapping patterns during embryonic development and in adult tissues of the mouse. In shared expression domains, the two ligands have redundant or different functions depending on the developmental context. An example for full redundancy is the maintenance of the crypt progenitor pool in the adult small intestine. *Dll1* and *Dll4* are coexpressed in crypt cells [25,26] and individual inactivation of either ligand has no effect on the crypt progenitor cell pool. However, simultaneous deletion of *Dll1* and *Dll4* leads to a complete loss of the proliferative crypt compartment and intestinal stem cells [27].

Conversely, in foetal arteries where both ligands are expressed in the vascular endothelium [26,28,29] inactivation of *Dll1* causes loss of NOTCH1 activation despite the presence of DLL4 [29] suggesting that DLL4 cannot compensate for the loss of DLL1 in fetal endothelial cells. In the adult thymus, *Dll1* and *Dll4* are both expressed in thymic epithelial cells [26,30]. Here, DLL4 is the essential Notch ligand required for T-lymphopoiesis [31] and T cell development is unaltered in mice lacking DLL1 in the thymic epithelium [32] suggesting that in this context DLL1 and DLL4 are functionally distinct. This conclusion is supported by *in vitro* studies showing that DLL1 and DLL4 differ with respect to their binding avidity to Notch receptors on thymocytes and to the steady-state cell surface levels required to induce T cell development, DLL4 being the more effective ligand [33,34] as well as by biochemical studies indicating a 10-fold higher Notch binding affinity of DLL4 than DLL1 [19]. Furthermore, DLL4 but not DLL1 can induce a fate switch in skeletal myoblasts and induce pericyte markers [35]. Collectively, these individual reports of context-dependent redundant and distinct functions of coexpressed DLL1 and DLL4 raise the questions of why DLL1 and DLL4 act equally in some processes but differently in others, which mechanism or factor causes their function to vary and whether they are similar enough to replace each other in domains where only one of both DLL ligands is endogenously expressed.

In early mouse embryos, expression of *Dll1* and *Dll4* is largely non-overlapping. *Dll1* is expressed in the paraxial mesoderm beginning at E7.5, in the central nervous system from E9 onwards and later on, at E13.5, in arterial endothelial cells [29,36]. Deletion of *Dll1* disrupts somite patterning and causes premature myogenic differentiation, severe haemorrhages and embryonic death after E11 [37,38]. *Dll4* is expressed in the vascular endothelium of arteries beginning at E8 [39] but not in the somite-generating presomitic mesoderm, somites or differentiating myoblasts. Inactivation of DLL4 results in severe vascular defects leading to embryonic death prior to E10.5 [39,40].

Here, we address the functional equivalence of DLL1 and DLL4 *in vivo* and *in vitro*. We analyse Notch signalling in mice that conditionally overexpress DLL1 or DLL4 on a *Dll1* null genetic background and in mice in which *Dll1* is replaced by *Dll4*, focussing on young embryos in which both Notch ligands have discrete endogenous expression domains. We show that DLL4 cannot replace DLL1 during somite segmentation but can partially replace DLL1 during myogenesis and fully replace DLL1 during maintenance of retinal progenitors. Cell culture assays that measure Notch activation by DLL1 or DLL4 demonstrate that DLL4 *trans*-activates Notch signalling similarly to DLL1 but *cis*-inhibits Notch signalling much more efficiently than DLL1, partly overruling the activation by interactions in *trans*. Consistent with these *in vitro* data, we observe dominant effects on segmentation by DLL4 ectopically expressed in the presomitic mesoderm (PSM). We propose that differential Notch *cis*-inhibition by DLL1 and DLL4 contributes to the observed tissue-dependent functional divergence of both paralogues, perhaps in combination with other factors not tested in this study.

## Results

### Mesodermal expression of DLL1 but not DLL4 rescues the *Dll1* knock-out somitogenesis phenotype

**Fig 1:**
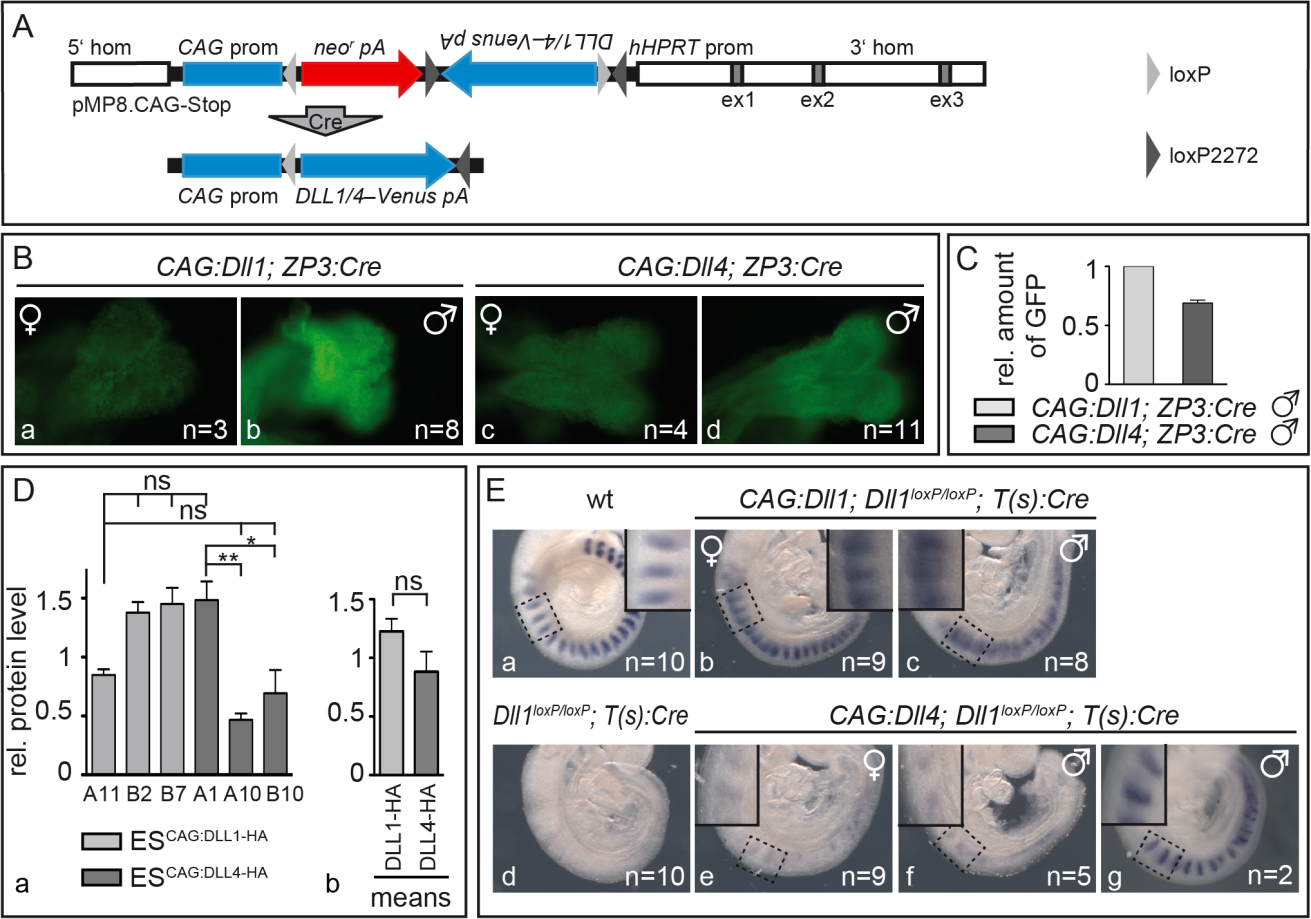
Mesodermally expressed CAG:DLL1 but not CAG:DLL4 functionally replaces endogenous DLL1 during somitogenesis. **(A)** Structure of unrecombined and recombined (bottom) pMP8.CAG-Stop/Dll vector for integration of Cre-inducible expression constructs into the *Hprt* locus. 5‘hom and 3‘hom, 5’ and 3’ homology regions from the *Hprt* gene for homologous recombination; ex (grey boxes), *HPRT* exons; *CAG* prom, *CAG* promoter to drive transgene expression; *neo* ^r^, neomycin phosphotransferase; *pA*, polyadenylation signal; *Dll1/4*–*Venus*, *Dll1* or *Dll4* ORF–joined to the reporter gene *Venus* by an *internal ribosomal entry site* (*IRES*); *hHPRT* prom, human *HPRT* promoter; light/dark grey triangles, *loxP*/*loxM* sites (in “flip excision” orientation); “Cre” arrow, Cre-mediated recombination. **(B)** Venus reporter expression in E8.5 *CAG*:*Dll1* and *CAG*:*Dll4* embryos indicated ubiquitous transgene activation after *ZP3*:*Cre*-mediated recombination. As expected, overall fluorescence in female embryos (a,c) was weaker than in male embryos (b,d) due to random X-chromosome inactivation. Numbers of embryos analysed are given in bottom right corner. **(C)** Quantification of Venus protein (*CAG*:*Dll1* set to one) by Western blot analysis of embryo lysates with anti-GFP antibodies (and anti-β-actin antibodies for normalisation) showed similar expression levels. **(D)** For direct comparison of DLL protein levels, we also integrated single copies of *Dll1* and *Dll4* labelled with C-terminal HA-tags following the strategy in (A) using recombined (active) constructs for electroporation of embryonic stem (ES) cells. Western blot analysis of three ES cell clones expressing either of these transgenes using anti-HA antibodies confirmed similar expression levels with expected mild clonal variations (a); means of all three *CAG*:*Dll1-HA* and all three *CAG*:*Dll4-HA* clones are shown in (b). **(E)** Cranial-caudal somite patterning visualised by whole mount *in situ* hybridisation of E9.5 embryos with an *Uncx4*.*1* probe showed an extensive rescue of somitogenesis plus ectopic Notch activation by CAG:DLL1 (b,c) but no appreciable rescue of somitogenesis by CAG:DLL4 (e,f). Insets in Ea-c and Ee-g show higher magnifications of the regions as indicated. Error bars represent standard error of the mean (SEM); ns, not significant; *, P<0.05; **, P<0.01.

In order to directly compare the activities of DLL1 and DLL4 *in vivo*, we generated mice that conditionally express either *Dll1* or *Dll4* under the CAG promoter from a single-copy transgene insertion in the same genomic locus. We employed an established system for integration of Cre-inducible expression constructs into the *Hprt* locus, the pMP8.CAG-Stop vector (Fig 1A; [41,42]). The unrecombined pMP8.CAG-Stop construct expresses neomycin phosphotransferase (*neo* ^*r*^) from the CAG promoter. Cre-mediated recombination of two *loxP* sites and two mutant *loxP2272* (*loxM*) sites [43] flips the gene of interest and excises *neo* ^*r*^ so that the recombined construct expresses the gene of interest from the CAG promoter. 5’ and 3’ homology regions from the *Hprt* gene enable homologous recombination of pMP8 constructs into the *Hprt* locus [44]. We cloned the *Dll1* and *Dll4* open reading frames into the pMP8.CAG-Stop vector, introduced both unrecombined (i.e. *neo* ^*r*^ expressing) constructs into *Hprt*-deficient E14TG2a ES cells and used homologous recombinant clones to produce transgenic mice with Cre-inducible *Dll1* or *Dll4* (alleles termed *CAG*:*Dll1* and *CAG*:*Dll4*).

To check activation of DLL1 and DLL4 in embryos, we induced ubiquitous expression of the *CAG*:*Dll1* and *CAG*:*Dll4* transgene by mating our mice with mice carrying a *ZP3*:*Cre* transgene that causes site-specific recombination during oogenesis [45]. We crossed *CAG*:*Dll1;ZP3*:*Cre* and *CAG*:*Dll4;ZP3*:*Cre* females with wildtype males to obtain embryos that overexpress *Dll1* or *Dll4* from the zygote stage on. The transgenes are transcribed bicistronically with an *IRES-Venus* (Fig 1A) whose expression marks cells in which Cre-recombination activated the transgene. As *Hprt* is located on the X chromosome, hemizygous male embryos expressed Venus ubiquitously whereas heterozygous female embryos showed mosaic expression due to random X-inactivation (Fig 1B). Analysis of embryo lysates on a Western blot with anti-GFP antibodies demonstrated *CAG*:*Dll1* and -*4* transgene activation at similar levels (Fig 1C; S1A Fig; S1 Table). To directly compare DLL1 and DLL4 protein levels, we generated embryonic stem cells expressing HA-tagged *Dll1* or *Dll4* from single copy insertions of in the *Hprt* locus. Western blot analysis of three independent clones for each ligand confirmed similar protein levels in all clones (average DLL1-HA, 1.23±0.33; average DLL4-HA, 0.88±0.53; Fig 1D; S1B Fig; S2 Table).

In order to test whether DLL4 can compensate for the loss of DLL1 in mesodermal tissues of early embryos, we mated mice to combine three different transgenes: a) *CAG*:*Dll1* or *-4* inducible transgenes; b) two (i.e. homozygous) floxed alleles of endogenous *Dll1* [Dll1loxP/loxP; 32] that get inactive upon recombination; and c) a *Cre* transgene expressed in the primitive streak driven by a promoter derived from brachyury [T(s):Cre; 46]. In offspring bearing the complete set of transgenes (identified by genotyping PCR; see Material and Methods), recombination by *T(s)*:*Cre* simultaneously inactivates endogenous *Dll1* and activates CAG:DLL1 or CAG:DLL4 expression in all mesoderm-derived tissues.

As expected, inactivation of *Dll1* throughout the mesoderm resulted in severe somite patterning defects characterised by loss of *Uncx4*.*1* expression (Fig 1Ed), a marker for caudal somite compartments [47,48] whose expression depends on Notch activation [46]. Expression of CAG:DLL1 in such *Dll1*-deficient embryos restored robust, largely regularly striped expression of *Uncx4*.*1*, which expanded into cranial somite compartments in most axial regions and particularly in hemizygous male embryos (Fig 1Eb and 1Ec). This rescue of somitogenesis demonstrates that expression of CAG:DLL1 (from the *Hprt* locus) is sufficient to substitute for the loss of endogenous DLL1; cranial expansion of *Uncx4*.*1* is reminiscent of ectopic Notch activity [46].

In contrast, expression of CAG:DLL4 in *Dll1*-deficient embryos restored only very weak and irregular expression of *Uncx4*.*1* and resembles *Dll1* ^*loxP/loxP*^ *;T(s)*:*Cre* embryos without CAG:DLL1 overexpression (Fig 1Ee and 1Ef). Only two out of 16 embryos of this genotype displayed regular *Uncx4*.*1* expression in the cranial most somites, which might reflect residual DLL1 activity perhaps due to delayed excision of endogenous *Dll1* (Fig 1Eg). The extensively defective segmentation in *Dll1* ^*loxP/loxP*^ *;T(s)*:*Cre* embryos with CAG:DLL4 overexpression directly shows a functional difference between DLL1 and DLL4 during early embryogenesis: DLL4 is not able to take over DLL1 function in the paraxial mesoderm during somite formation. Weak and irregular *Uncx4*.*1* expression in some of these embryos suggest Notch activation at low levels.

### Mice expressing DLL4 in place of DLL1 from the *Dll1* locus reveal divergent function during somitogenesis

**Fig 2:**
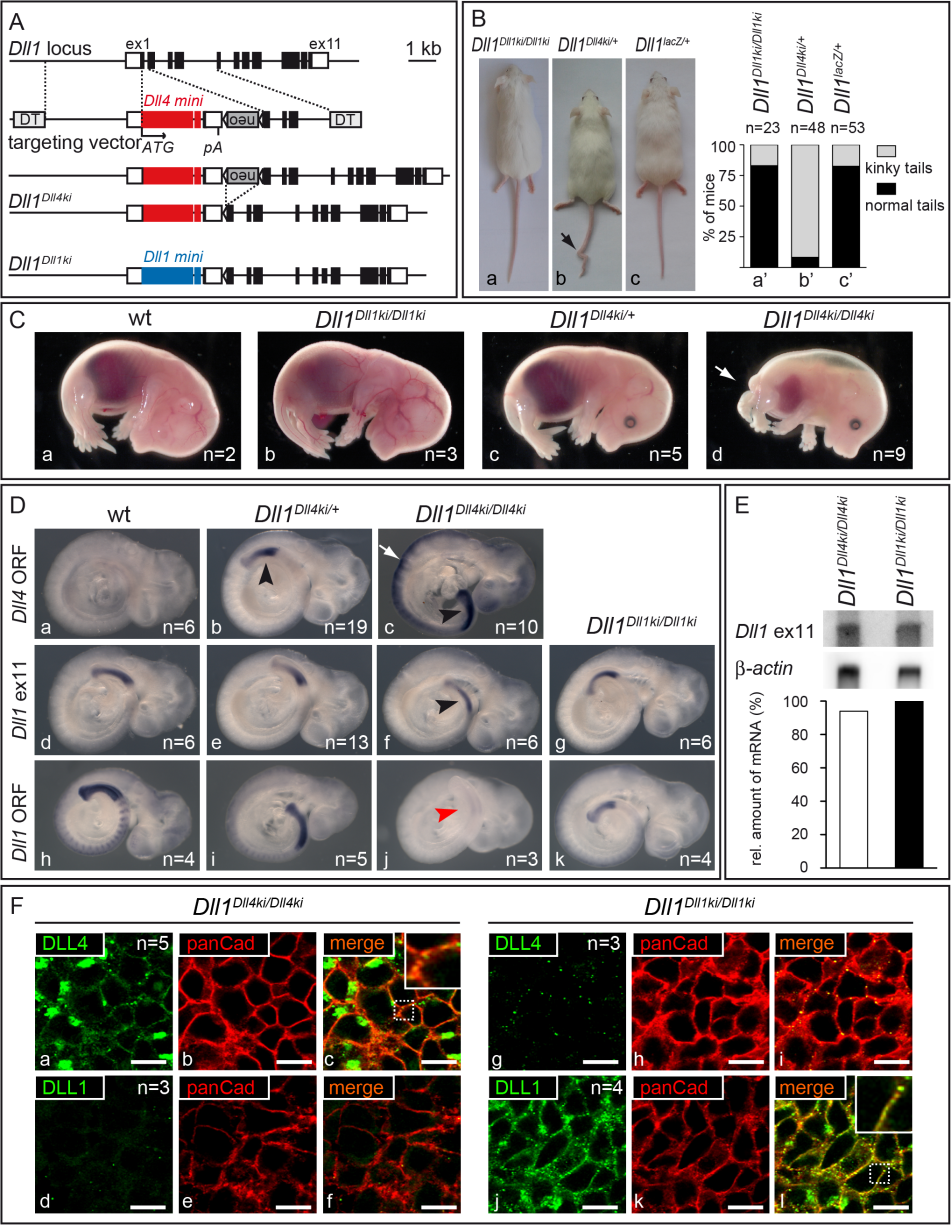
Generation of *Dll1* ^*Dll4ki*^ mice that express *Dll4* instead of *Dll1* in the endogenous *Dll1* domains. **(A)** Targeting strategy to insert a *Dll4* mini gene into the *Dll1* locus. The *Dll1* locus contains 11 exons depicted as black boxes (UTRs as white boxes). The targeting construct is comprised of the *Dll4* mini gene [*Dll4* cDNA from start codon (ATG) in exon 1 to exon 9 (large red box), *Dll1* intron 9, *Dll4* exon 10 (small red box), *Dll1* intron 10 and *Dll1* exon 11 that encodes only the terminal valine conserved between *Dll1* and *Dll4* followed by STOP codon and 3‘UTR], a floxed *neo* ^r^ cassette, homology regions for integration between *Dll1* start codon and exon 2, and flanking diphtheria toxin genes (DT); insertion of the mini gene is expected to disrupt expression of *Dll1*. *neo* ^r^ is removed by Cre-recombination. The resulting *Dll1* *^Dll4ki^* allele and the *Dll1* *^Dll1ki^* control are shown below (blue boxes, *Dll1* mini gene). **(B)** Heterozygous adult *Dll1* *^Dll4ki^* mice frequently (89%) displayed a kinky tail (arrow in b) but looked otherwise normal. **(C)** Heterozygous E15.5 *Dll1* *^Dll4ki^* foetuses (c) were indistinguishable from wildtype (wt; a) and homozygous *Dll1* *^Dll1ki^* (b) foetuses while all homozygous *Dll1* *^Dll4ki^* foetuses (d) displayed shortened body axes and large oedemas. **(D)** *Dll1* and *Dll4* expression in *Dll1* *^Dll4ki^* and *Dll1* *^Dll1ki^* embryos visualised by whole mount *in situ* hybridisation of E9.5 embryos of the indicated genotype with a *Dll4* ORF, *Dll1* ex11 (recognises transcripts from both mini genes) and *Dll1* ORF probe confirmed that *Dll4ki* alleles expressed *Dll4* but not *Dll1* in *Dll1* expression domains (here the PSM, arrowheads). a-c were stained in parallel and colour development was stopped before endogenous *Dll4* expression [49] and background became visible. Homozygous *Dll1* *^Dll4ki^* embryos show strong expression in neuroectoderm (white arrow in c; not visible in the weaker staining with *Dll1* ex11 probe in f). **(E)** Northern blot analysis of homozygous *Dll1* *^Dll4ki^* and *Dll1* *^Dll1ki^* E11.5 embryos, 2 μg polyA(+)-RNA loaded per lane, hybridised with 3‘UTR (*Dll1* ex11) and β-actin probes; quantification of transgene signals relative to actin is shown at the bottom and indicates similar expression levels. **(F)** Visualisation of DLL1 and DLL4 expressed in the PSM of homozygous *Dll1* *^Dll4ki^* (a-f) and *Dll1* *^Dll1ki^* E9.5 embryos (g-l) using specific anti-DLL1 and anti-DLL4 antibodies. Co-staining with anti-panCadherin antibodies, which mark the plasma membrane, confirms that transgenic DLL4 and DLL1 predominantly localise to the cell surface (c,l). The lack of DLL1 signal in *Dll1* *^Dll4ki^* (d) and of DLL4 signal in *Dll1* *^Dll1ki^* PSMs (g) confirm the specificity of stainings. Both in anti-DLL4 and anti-DLL1 antibody stainings of PSMs, we observed spots of high signal intensity that may result from accumulation of ligands at these sites and that had also been observed in wildtype PSMs stained with anti-DLL1 antibodies [21]. Scale bars, 10 μm; insets show magnifications of the dotted boxes in c,l.

To further investigate to which degree DLL4 can compensate for the loss of DLL1 during somite patterning and in other developmental contexts, we generated mice that express DLL4 from the *Dll1* locus instead of endogenous DLL1. To replace endogenous *Dll1* with *Dll4*, we applied a knock-in strategy inserting a *Dll4* mini gene into the first and second exons of *Dll1* (Fig 2A). Production of DLL4 protein of the correct size from the *Dll4* mini gene was confirmed by Western blot analysis of lysates of CHO cells transiently expressing the *Dll4* mini gene (S2 Fig). We generated mice carrying the *Dll4* mini gene in the *Dll1* locus, referred to as *Dll1* ^*Dll4ki*^. As a control, we used the analogous knock-in of a *Dll1* mini gene into the *Dll1* locus (Fig 2A bottom; *Dll1* ^*tm2Gos*^, here referred to as *Dll1* ^*Dll1ki*^), which was identical to the *Dll4* mini gene with regard to its exon/intron structure, intron sequences and the 5' and 3' UTRs but encoded DLL1. Homozygous *Dll1* ^*Dll1ki*^ mice were viable and fertile and appeared phenotypically normal indicating that the *Dll1* mini gene can functionally substitute the endogenous *Dll1* gene [37].

Heterozygous *Dll1* ^*Dll4ki/+*^ mice (containing one endogenous copy of *Dll1* and one copy of *Dll4ki*) were viable and fertile and showed no obvious phenotype except for kinky tails (Fig 2Ba–2Bc, arrow; penetrance 89%; n = 48), a phenotype indicative of irregular somitogenesis rarely observed in *Dll1* ^*Dll1ki*^ homozygotes or *Dll1* null (*Dll1* ^*lacZ*^) heterozygotes (penetrance 15%; n = 23 and 53, respectively; Fig 2Ba’–2Bc’). In contrast to homozygous *Dll1* ^*Dll1ki*^, no homozygous *Dll1* ^*Dll4ki*^ mice were obtained after birth. At E15.5, *Dll1* ^*Dll4ki*^ homozygotes exhibited short body axes, truncated tails and were oedematic (Fig 2C; arrow points at tip of tail) resembling foetuses with severely reduced DLL1 function [37].

Correct expression of *Dll4* in the presomitic mesoderm (PSM) of *Dll1* ^*Dll4ki*^ embryos was confirmed by *in situ* hybridisation using probes specific for the *Dll4* ORF or the 3‘UTR (*Dll1* exon 11) common to *Dll1* ^*Dll1ki*^ and *Dll1* ^*Dll4ki*^ alleles (Fig 2Db, 2Dc and 2Df, black arrowheads). *In situ* hybridisation with a specific *Dll1* ORF probe confirmed the absence of *Dll1* transcripts in *Dll1* ^*Dll4ki*^ homozygotes (Fig 2Dj, red arrowhead). Homozygous *Dll1* ^*Dll4ki*^ embryos showed strong expression of *Dll4* in the neural tube (Fig 2Dc, white arrow), reflecting activation of the *Dll1* promoter in this region [50,51]. Northern blot analysis of *Dll1* ^*Dll1ki*^ and *Dll1* ^*Dll4ki*^ homozygous embryos indicated equal levels of transcription of the transgenes (Fig 2E). In *Dll1* ^*Dll4ki*^/^*Dll4ki*^ embryos, ectopic DLL4 protein was detected at the plasma membrane of PSM cells (Fig 2Fa–2Fc). Likewise, DLL1 protein was detected at the surface of PSM cells in homozygous *Dll1* ^*Dll1ki*^ embryos (Fig 2Fj–2Fl), confirming that DLL4 and DLL1 protein is generated from their mini genes and targeted to the plasma membrane *in vivo*. Taken together, these data show that *Dll1* ^*Dll4ki*^ mice indeed express *Dll4* instead of *Dll1* from the *Dll1* locus at comparable levels and confirm our previous observation that DLL4 is unable to support proper mouse development in the absence of endogenous DLL1.

**Fig 3:**
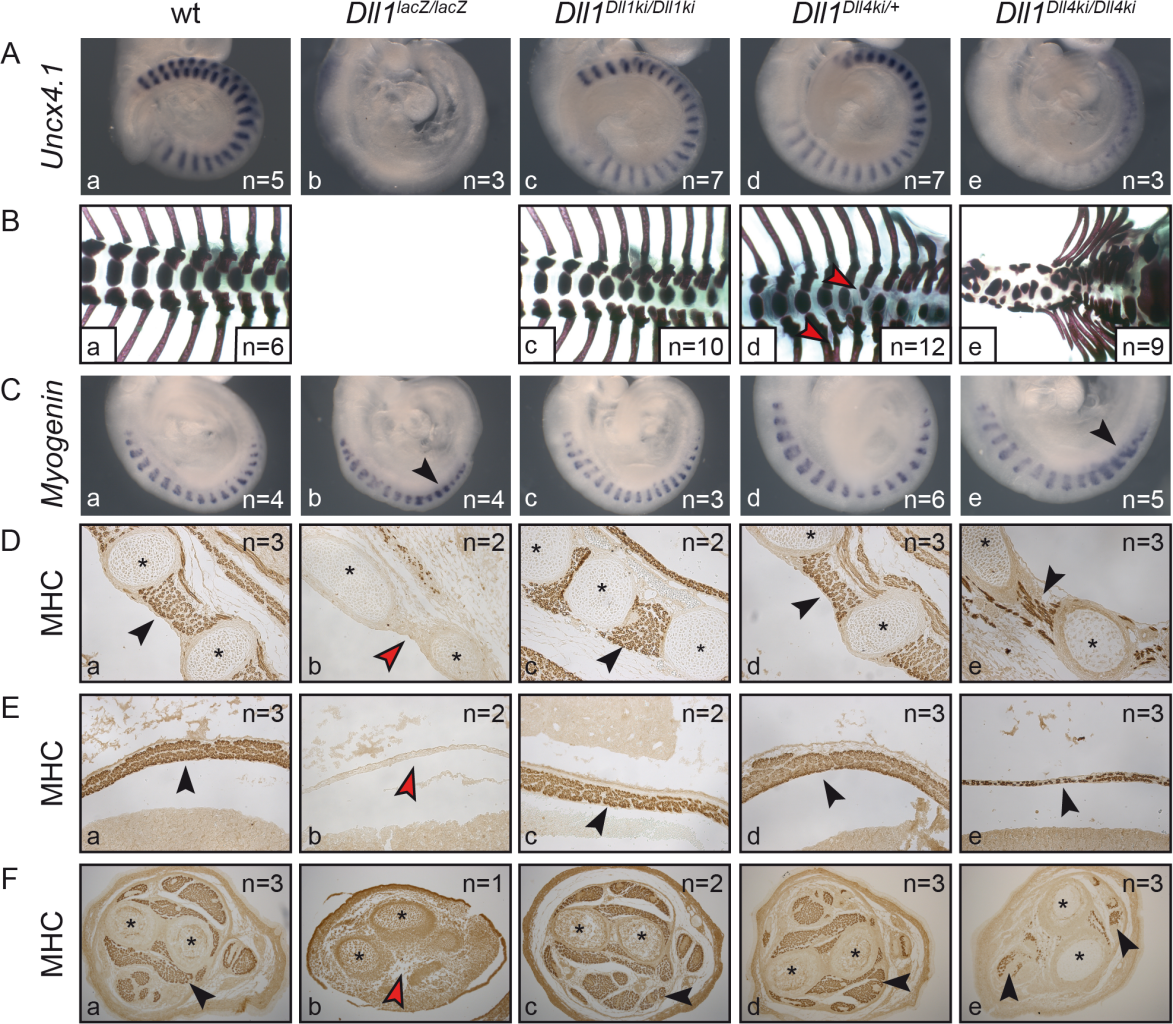
Homozygous *Dll1* *^Dll4ki^* mice fail to generate proper somites and form reduced skeletal muscle tissue. Examination of *Dll1*-dependent (A,B) somitogenesis and (C-F) myogenesis in (a) wildtype, (b) *Dll1* *^lacZ/lacZ^*, (c) *Dll1* *^Dll1ki/Dll1ki^*, (d) *Dll1* *^Dll4ki/+^* and (e) *Dll1* *^Dll4ki/Dll4ki^* embryos or foetuses. **(A)** *Uncx4*.*1 in situ* hybridisation of E9.5 embryos. **(B)** Skeletal preparations of E18.5 foetuses (*Dll1* *^lacZ/lacZ^* foetuses do not survive until E18.5; red arrowheads indicate fused ribs or hemivertebrae in heterozygous *Dll1* *^Dll4ki^* skeletons in d). **(C)** *Myogenin in situ* hybridisation to visualise differentiating skeletal muscle cells in myotomes of 17–18 somite stage embryos. **(D, E,F)** Anti-myosin heavy chain (MHC)-antibody staining of sectioned E15.5 foetuses showing intercostal muscles (D), the diaphragm (E), and muscles in the cross-section of forelimbs (F); black arrowheads indicate examples of muscle tissue, red arrowheads show lack of muscle tissue; asterisks label ribs (D) or bones of the forelimb (F).

Cranial-caudal somite patterning critically depends on DLL1-mediated Notch signalling [38,46,52]. We analysed if DLL4 can functionally replace DLL1 in this process in homozygous *Dll1* ^*Dll4ki*^ embryos. Unlike embryos that contained at least one wildtype or *Dll1* ^*Dll1ki*^ allele, homozygous *Dll1* ^*Dll4ki*^ embryos displayed severely reduced and irregular *Uncx4*.*1* expression (Fig 3A), which indicates disrupted somite patterning and reduced Notch activity in the PSM due to the inability of DLL4 to replace DLL1. Consistent with defective somite formation and the shortened body axis observed in E15.5 foetuses, *Dll1* ^*Dll4ki/Dll4ki*^ axial skeletons were severely disorganised (Fig 3B). Therefore, expression of DLL4 from the *Dll1* locus does not cause a significant rescue of the *Dll1* somitogenesis phenotype.

Remarkably, as anticipated by the kinky tail phenotype of heterozygous *Dll1* ^*Dll4ki/+*^ adults (Fig 2B), E18.5 *Dll1* ^*Dll4ki/+*^ skeletons reveal fusions of dorsal ribs and malformations of individual vertebrae in various regions of the vertebral column (e.g. Fig 3Bd, red arrowheads; 8/12 E18.5 *Dll1* ^*Dll4ki/+*^ skeletons displayed apparently irregular vertebrae). Additional examination of seven *Dll1* ^*Dll4ki/+*^ adult skeletons uncovered fused ribs and/or irregular segments in the tail of all preparations (S3 Fig). A single *Dll1* allele is sufficient to support regular segmentation and *Dll1* ^*lacZ/+*^ mice form essentially normal skeletons [53]. Our consistent finding of skeletal irregularities in *Dll1* ^*Dll4ki/+*^ mice indicates subtle disturbances during segmentation and suggests that the *Dll1* ^*Dll4ki*^ allele has a dominant effect on segmentation.

### DLL4 can partially substitute for DLL1 during myogenesis and fully replace DLL1 during early retinal development

Processes other than somitogenesis in the developing embryo that depend on DLL1–Notch signalling include myogenesis [37] and retinal development [51]. Embryos lacking DLL1 display excessive differentiation of myoblasts, which exhausts the progenitor pool and leads to severely reduced or absent skeletal muscles [37]. Homozygous E9.5 *Dll1* ^*Dll4ki*^ embryos showed transient upregulation of the myocyte marker *Myogenin* [54] as also observed in homozygous *Dll1* ^*lacZ*^ embryos (Fig 3C, arrowheads; [37]). At E15.5, they had significantly less skeletal muscle tissue than wildtype or homozygous *Dll1* ^*Dll1ki*^ foetuses but clearly more skeletal muscle tissue than *Dll1* null mutants (*Dll1* ^*lacZ*^) as shown for the intercostal muscles, the diaphragm and forelimbs by anti-MHC antibody staining of sectioned foetuses (Fig 3D–3F, arrowheads). These results indicate that DLL4 can partially substitute DLL1 during muscle cell differentiation and *Dll1* ^*Dll4ki*^ behaves like a hypomorphic *Dll1* allele.

**Fig 4:**
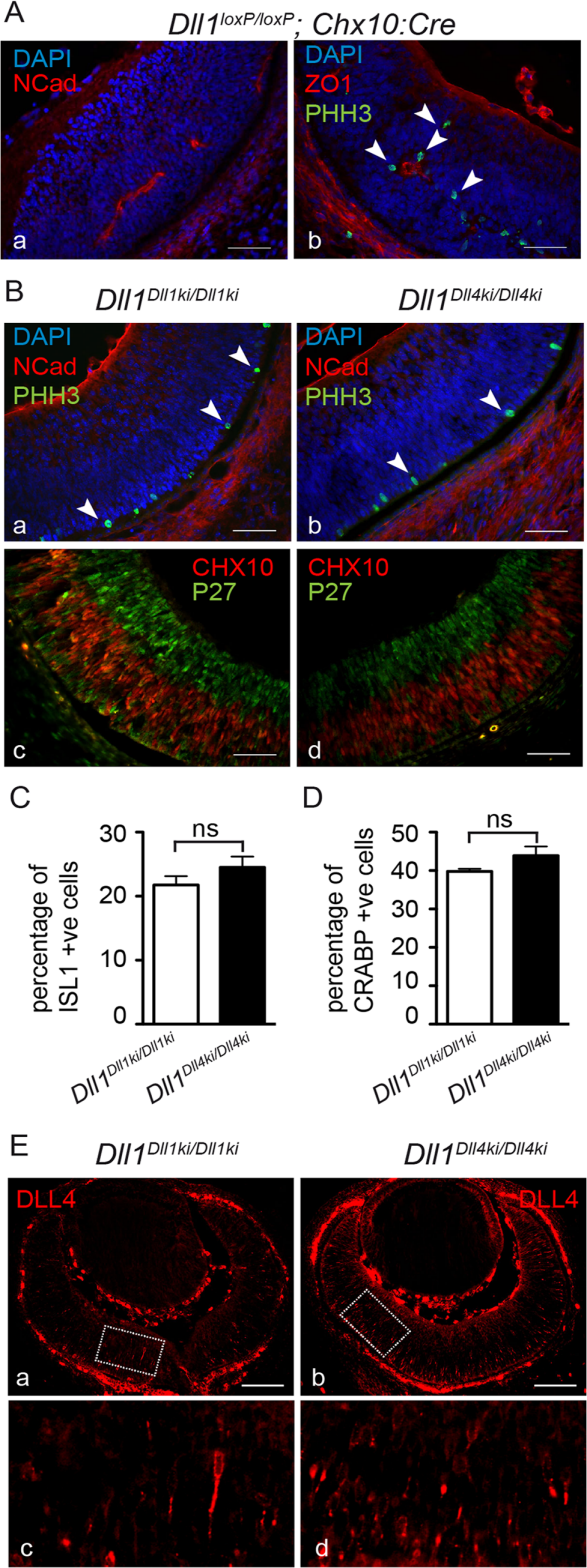
DLL4 expressed from the *Dll1* locus rescues DLL1 loss-of-function in the retina. **(A)** *Dll1* null mutant retinas show epithelial disruption with formation of polarised rosettes in which the apical markers N-Cadherin (NCad, a) and ZO-1 (ZO1, b) are abnormally present at the central lumen. Ectopic proliferating progenitors, labelled with PHH3 (b, arrowheads), are located close to the apical lumen of these rosettes. **(B)** In contrast, the neuroepithelium of homozygous *Dll1* *^Dll1ki^* and *Dll1* *^Dll4ki^* embryos is correctly organised without rosettes, and N-Cadherin shows the normal apical localisation close to the retinal pigmented epithelium (a,b). Mitotic progenitors (PHH3+) are only detected at the apical region of the neuroepithelium (a,b arrowheads). A normal stratification of CHX10+ progenitors and P27+ differentiating neurons is also observed (c,d). **(C, D)** E13.5 homozygous *Dll1* *^Dll1ki^* and *Dll1* *^Dll4ki^* retinas show no significant difference in the number of ISL1+ RGCs (C) and CRABP+ amacrine cells (D). Cells immunopositive for Islet-1 and Crabp were counted and related to the total number of cells in the retina (DAPI+). Percentages are shown as mean ± SEM; ns, not significant. **(E)** Expression of DLL4 in homozygous *Dll1* *^Dll1ki^* (a,c) and in homozygous *Dll1* *^Dll4ki^* (b,d) E13.5 retinas as detected by an anti-DLL4 antibody. (c) and (d) are magnifications of (a) and (b), respectively. Endogenous plus transgenic DLL4 is expressed in more cells in *Dll1* *^Dll4ki/Dll4ki^* as compared to endogenous DLL4 expression in *Dll1* *^Dll1ki/Dll1ki^* while signal strength is similar. Scale bars are 50 μm in (A, B) and 100 μm in (E).

In the embryonic neural retina, *Dll1* and *Dll4* are sequentially expressed and can both function to maintain proliferating progenitors, while they have different functions in retinal fate diversification [51,55]. In contrast to myogenesis, DLL4 can fully replace DLL1 function in maintaining neuronal progenitors in the embryonic retina. Whereas *Dll1* mutants show a striking disruption of the retinal neuroepithelium with formation of rosettes (Fig 4A), due to premature differentiation of retinal progenitors [51], both *Dll1* ^*Dll1ki/Dll1ki*^ and *Dll1* ^*Dll4ki/Dll4ki*^ retinas have a normal neuroepithelial organisation with a clear stratification of Chx10+ progenitors and p27+ differentiating neurons (Fig 4B). Moreover, we find that similar numbers of early born retinal neurons [retinal ganglion cells (RGCs) and amacrine cells] are present in *Dll1* ^*Dll1ki*^ and *Dll1* ^*Dll4ki*^ retinas (Fig 4C and 4D; n≥4 retinal sections), confirming that DLL1 and DLL4 functions are interchangeable in regulating early retinal neurogenesis. We have further analysed DLL4 expression in *Dll1* ^*Dll4ki/Dll4ki*^ retinas and found it recapitulates the broader *Dll1* expression pattern, with the transgenic protein expressed at similar levels as endogenous DLL4 in the retinal neuroepithelium (compare Fig 4Ea–4Ec with 4Eb–4Ed). Together, these results offer further evidence that the *Dll4* transgene is fully functional in *Dll1* ^*Dll4ki/Dll4ki*^ embryos. The extent of the functional equivalence of DLL1 and DLL4 depends on the developmental context.

### DLL1 and DLL4 activate Notch similarly *in vitro*

**Fig 5:**
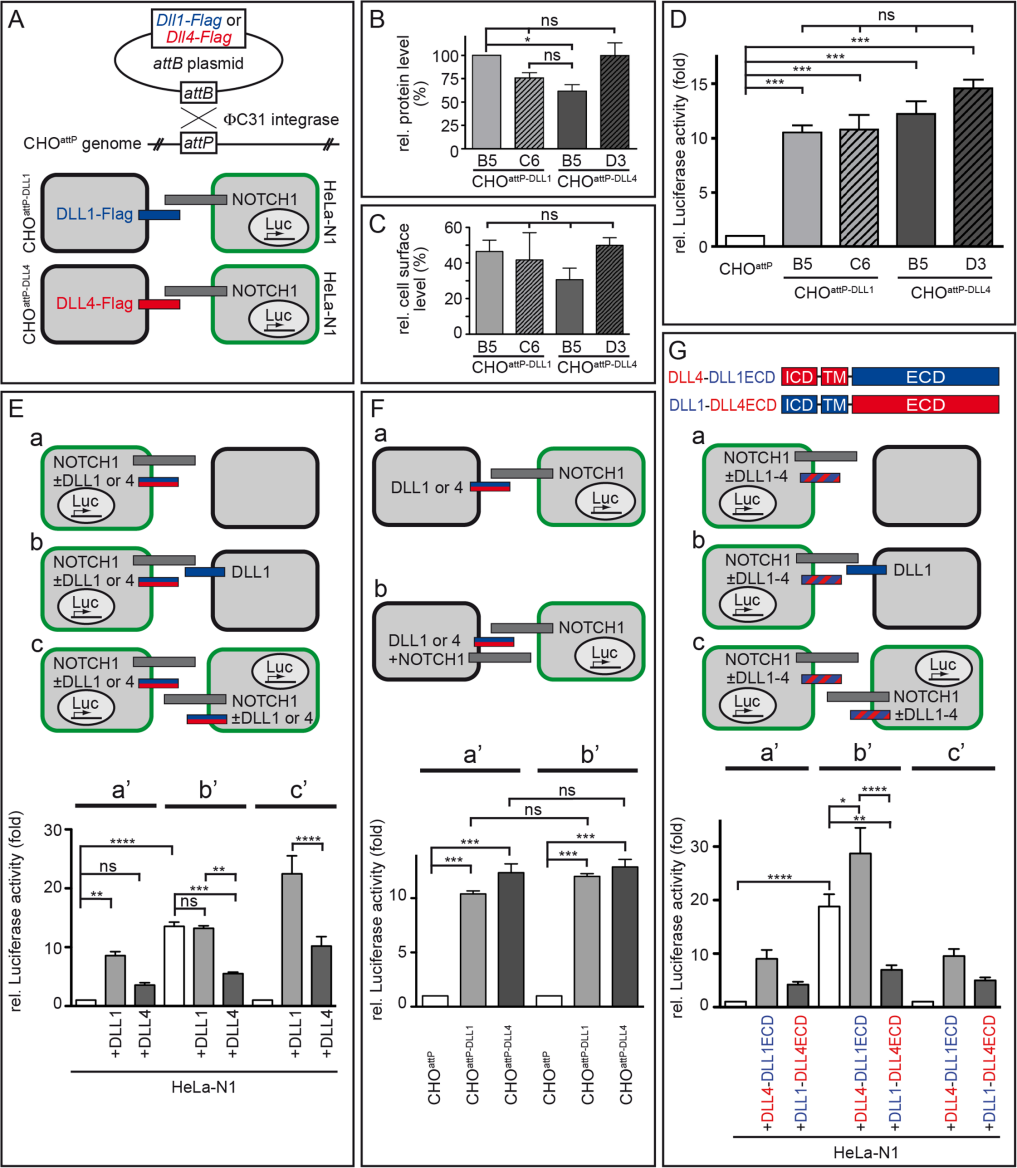
DLL1 and DLL4 *trans*-activate Notch with similar efficiency, but only DLL4 is an effective *cis*-inhibitor. **(A)** Flag-tagged *Dll1* and *Dll4* ORFs were inserted into a randomly integrated *attP* site in CHO^attP^ cells mediated by ΦC31 site-directed recombination (upper part). Resulting cells were used in Notch-activation assays in combination with HeLa-N1 cells as schematically shown below (DLL1 depicted as blue bar; DLL4, red; NOTCH1, grey; HeLa-N1 cells are encircled in green). **(B)** Quantification of DLL1-Flag and DLL4-Flag in two independent CHO^attP-DLL1^ (B5, C6) and CHO^attP-DLL4^ (B5, D3) cell lines by Western blot analysis of cell lysates with anti-Flag and anti-β-actin (for normalisation) antibodies showed similar protein levels. **(C)** Surface biotinylation assays demonstrated equal surface representation of DLL1 and DLL4 on CHO^attP^ cells. **(D)** Notch *trans*-activation assays by co-culture of HeLa-N1 cells containing an RBP-Jκ:Luciferase reporter with CHO^attP-DLL1^ or CHO^attP-DLL4^ cells. All DLL1 and DLL4 clones activated Notch similarly, DLL4 being a slightly more efficient activator (compare with similar experiment in S6A and S6G Fig). **(E)** Notch *trans*-activation and *cis*-inhibition assays by culturing HeLa-N1 cells untransfected or transiently transfected with *Dll1* or *Dll4* expression constructs with or without CHO^attP^ or CHO^attP-DLL1^ cells as indicated (a-c). Co-culture conditions a, b and c correspond to Luciferase measurements a’, b’ and c’, respectively. Results show *cis*-inhibition by DLL4 but not DLL1; for details see main text. **(F)** *trans*-Activation assays (a) without and (b) with NOTCH1 receptor expression in the signal sending CHO cell to test if NOTCH1 *cis*-inhibits the ligand activity of DLL1 or DLL4. No *cis*-inhibitory effect on either ligand was observed (columns a‘ and b‘ correspond to assay conditions a and b, respectively). **(G)** *trans*-Activation and *cis*-inhibition assays using chimeric DLL1-DLL4 proteins (G top; depicted as red and blue striped bars in a-c). HeLa-N1 cells were transiently transfected with no or DLL4-DLL1ECD or DLL1-DLL4ECD expression constructs and cultured as indicated (a-c). Under all three conditions, a strong *cis*-inhibitory activity was detected only for DLL1-DLL4ECD (columns a‘, b’ and c‘ correspond to schemas a, b and c, respectively). Error bars represent SEM; ns, not significant; *, P<0.05; **, P<0.01; ***, P<0.001; ****, P<0.0001.

To investigate the functional difference between DLL1 and DLL4 *in vitro*, we performed co-culture experiments by mixing cells expressing NOTCH1 receptor or DLL ligands and measured Notch activation with a reporter in the receptor-expressing cells. Specifically, we used HeLa cells that express both the NOTCH1 receptor (stable HeLa-N1 cells; [10]) and a transient Notch activity reporter based on an RBP-Jk promoter-driven Luciferase [56] with CHO cells stably expressing Flag-tagged DLL1 or DLL4 ligands. To ensure comparability of results, we integrated single copies of *Dll1* or *Dll4* ORFs under the control of the CMV promoter into the identical genomic locus of CHO cells by adopting a site-directed *attP/attB* recombination system (Fig 5A top; S4 Fig; [57]). We established CHO cells with a pre-inserted, randomly integrated single *attP* site (termed CHO^attP^; uniqueness of this *attP* site was confirmed by Southern blot analysis; S4A and S4B Fig) and recombined *Dll1* or *Dll4* ORFs into this site (cell lines termed CHO^attP-DLL1^ and CHO^attP-DLL4^; Fig 5A bottom left). Consistent with the expression from the same genomic locus, independent CHO^attP-DLL1^ (B5, C6) and CHO^attP-DLL4^ (B5, D3) clones expressed DLL1 and DLL4 protein at similar levels (Fig 5B; n = 4 lysates of each clone; S5A Fig, S3 Table, S5B Fig, S4 Table) and cell surface representation of DLL1 and DLL4 was similar in all lines (~40%; Fig 5C; n≥3 biotinylation assays; S5C and S5D Fig, S5 Table). Likewise, half-lives of DLL1 and DLL4 proteins were similar, DLL4 being slightly more stable (S5E and S5F Fig).

Co-culture of HeLa-N1 with either CHO^attP-DLL1^ or CHO^attP-DLL4^ (schematically shown in Fig 5A bottom) led to a >10-fold increase of Notch activity as compared to co-cultures of HeLa-N1 with CHO^attP^ cells that did not express transgenic DLL1 or DLL4 (Fig 5D; n = 3) confirming that all transgenes were functional. DLL4 trended to activate Notch more strongly than DLL1 (including clone CHO^attP-DLL4^ B5 whose protein level was slightly reduced in Fig 5B); the difference between individual clones was not statistically significant in these experiments and partly significant in similar experiments with other clones (S6A and S6G Fig).

Next, we tested whether coexpression of further factors (LFNG, JAG1) in our cell culture system differently alters Notch activation by DLL1 or DLL4 and thereby provides a plausible explanation for the distinct phenotypes. The glycosyltransferase LUNATIC FRINGE (LFNG), which is expressed in the PSM, is able to modify NOTCH in the *trans*-Golgi [58,59] and thereby modulates receptor activation. The Notch ligand JAG1 is expressed in forming somites [60,61] and can act as a competitive inhibitor of DLL ligands [62,63]. We performed co-culture assays with HeLa-N1 cells transiently expressing LFNG-HA (S6A–S6C Fig) or with CHO^attP-DLL1^ and CHO^attP-DLL4^ cells coexpressing JAG1 (S6D–S6F Fig) and found no statistically significant changes in Notch activation.

Also, different glycosylation patterns of the ligands’ extracellular domain could contribute to differences in their activity. To test this possible influence, we treated co-cultures with tunicamycin to prevent N-glycosylation. Blocking N-glycosylation reduced the activity of DLL4 in cultured cells significantly, but not below DLL1 activity (S6G and S6H Fig), suggesting that distinct N-glycosylation is an unlikely cause for the observed differences between both ligands. Collectively, our results do not reveal a difference in the *trans*-activation potential of DLL1 and DLL4 that could explain the different segmentation phenotypes of our transgenic DLL1- or DLL4-expressing mice.

### DLL4, but not DLL1, is a strong *cis*-inhibitor of Notch signalling

We modified the co-culture assay by (transiently) expressing the ligands in the HeLa-N1 cells instead of in the CHO cells (Fig 5E, S7 Fig). In this setting, DLL ligands (expressed in HeLa-N1 cells) can *trans*-activate Notch in neighboring HeLa-N1+DLL cells (schematically shown in Fig 5Ec or in detail in S7A Fig); in addition, they can interact with Notch expressed in the same cell, i.e. in *cis*. When co-culturing HeLa-N1 cells expressing DLL1 with empty CHO cells (Fig 5Ea, S7 Fig), activation of Notch signalling was significantly increased as compared to a co-culture of HeLa-N1 cells expressing no transgenic DLL ligand with empty CHO cells (Fig 5Ea’; compare light grey bar “+DLL1” and white bar; n = 6; numbers are normalised to white bar). Intriguingly, in co-cultures of HeLa-N1 cells expressing DLL4 (with empty CHO cells), Notch activation was significantly lower than with DLL1 (Fig 5Ea’; compare dark grey bar “+DLL4” and light grey bar “+DLL1”; n = 6). Given the similar *trans*-activation potential of DLL1 and DLL4 (Fig 5D; S6A and S6G Fig), a likely explanation for the different levels of Notch activation is a higher *cis*-inhibitory potential of DLL4 than of DLL1.

In order to facilitate the analysis of *cis*-inhibition, we repeated the experiments shown in Fig 5Ea and 5Ea’ with the only modification of expressing a DLL ligand in the CHO cells (CHO^attP-DLL1^) so as to enhance the level of Notch activation (Fig 5Eb and 5Eb’). In the experiments both without any transgenic DLL ligand in HeLa-N1 and with DLL1 in HeLa-N1, co-culture with CHO^attP-DLL1^ cells caused a >10-fold increase of Notch activation. In the HeLa-N1 cells with DLL4, Notch activation was significantly less, i.e. about 5-fold increased (Fig 5Eb; n = 6; all numbers in Fig 5Ea’ and 5Eb’ are normalised to the left bar in a’, i.e. HeLa-N1 without transgenic DLL co-cultured with empty CHO cells, set to 1). These results support *cis*-inhibition of Notch by DLL4 resulting in a strong reduction of net Notch activation. In contrast, DLL1 does not *cis*-inhibit Notch in this assay (no significant difference between white and light grey bar in Fig 5Eb’).

To approximate the setting of the embryonic cranial PSM in which every cell expresses both DLL1 and NOTCH1 [64], we also analysed pure cultures of HeLa-N1 cells expressing either no transgenic DLL or (transient) DLL1 or DLL4 (Fig 5Ec). Expression of DLL1 enhanced Notch activation ~15-fold whereas expression of DLL4 increased Notch activation only <5-fold which was not significantly different from HeLa-N1 cells without transgenic ligand (Fig 5Ec’; n = 6; numbers in Fig 5Ec’ are normalised to the left bar in 5Ec, i.e. culture of HeLa-N1 without transgenic DLL, set to 1). These data show that *cis*-inhibition by DLL4 partially overrides *trans*-activation, and reduces Notch activation to <30% in an *in vitro* setting modeling the arrangement of ligand and receptor molecules in the PSM.

A conceivable alternative explanation of our *in vitro* results, which show attenuated Notch signalling when NOTCH and DLL4 are coexpressed, could be a reciprocal mechanism, i.e. *cis*-inhibition of DLL4 by NOTCH1 [18]. To test this possibility, we modified our first Notch activation assay (Fig 5A bottom, 5D) by transiently coexpressing NOTCH1 (NOTCH1deltaC, see Methods) in CHO^attP-DLL1^ and CHO^attP-DLL4^ cells. In co-cultures with HeLa-N1 cells containing the reporter (Fig 5Fa and 5Fb, S8 Fig), both ligands activated NOTCH in HeLa-N1 >10-fold irrespective of the presence of NOTCH1 in the CHO cells (Fig 5Fa’ and 5Fb’; n = 3) and we measured no significant difference between *trans*-activation by DLL1 or DLL4 as before (Fig 5D). These data indicate that NOTCH1 does not *cis*-inhibit its ligands.

**Fig 6:**
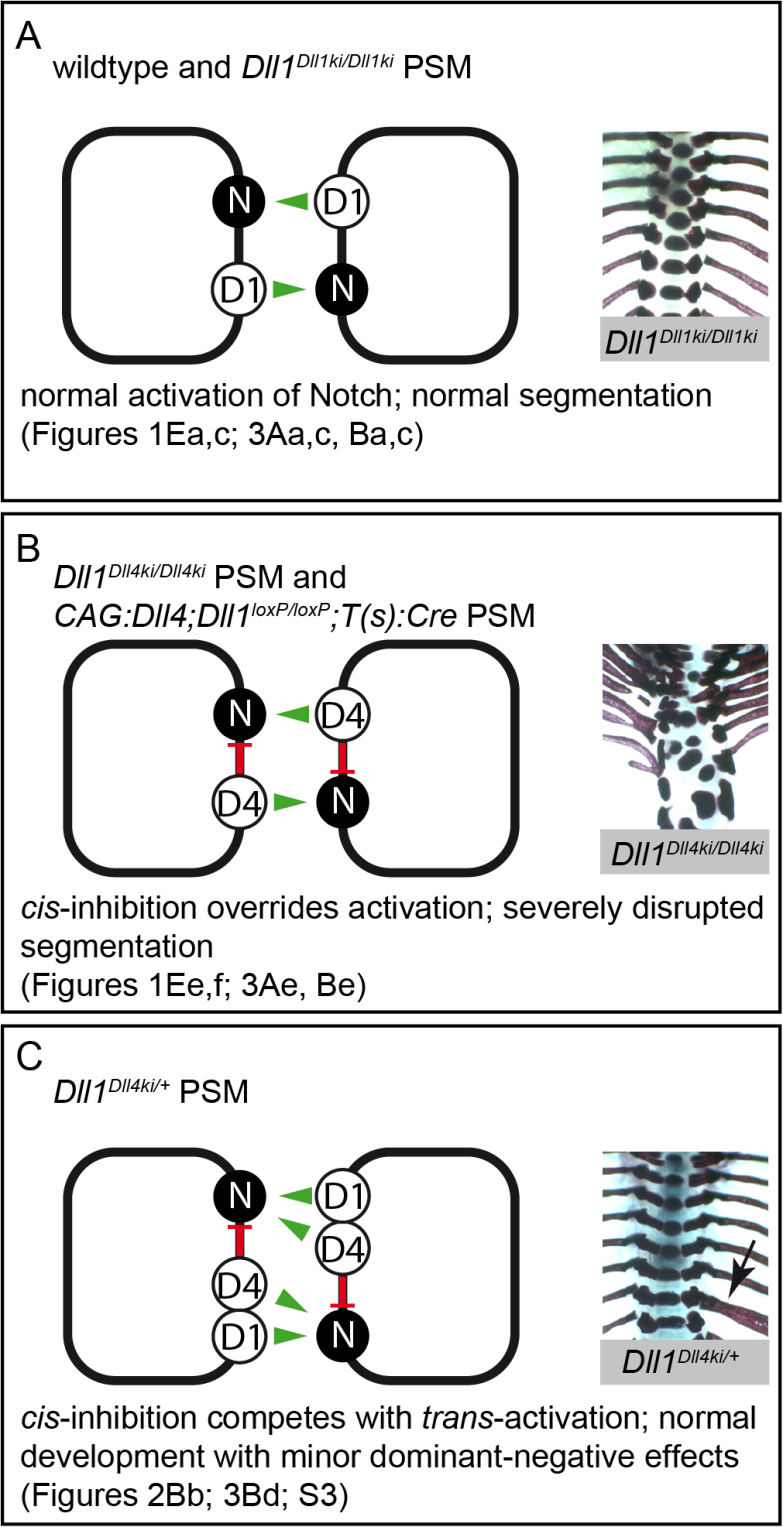
Model of Notch signalling in the PSM triggered by DLL1 and ectopic DLL4. Summary combining our *in vivo* and *in vitro* data in three different genetic scenarios (A-C); *trans*-activation (green arrows) and *cis*-inhibition (red bars) in cells of the PSM are schematically depicted on the left, representative skeletal preparations to visualise the outcome of somitogenesis are shown on the right; references to Figs. in this paper are given below. **(A)** In wildtype and *Dll1* *^Dll1ki/Dll1ki^* PSMs, endogenous or transgenic DLL1 (D1) *trans*-activates Notch (N) signalling and results in a regularly segmented axial skeleton. **(B)** In our *in vitro* assays, DLL4 (D4) *trans*-activates Notch with similar efficiency as DLL1 but has an additional strong *cis*-inhibitory effect on Notch signalling that partially overrides *trans*-activation. The reduced net Notch activation in *Dll1* *^Dll4ki/Dll4ki^* and *CAG*:*Dll4;Dll1* *^loxP/loxP^* *;T(s)*:*Cre* PSMs is insufficient to support normal segmentation. **(C)** When both DLL1 and DLL4 are expressed (*Dll1* ^Dll4ki/+^ PSM), *cis*-inhibition by DLL4 plays a relatively smaller role, the resulting axial skeletons are mostly regular. However, *cis*-inhibition by DLL4 reduces the robustness of Notch signalling resulting in minor malformations (arrow indicates a misplaced rib), which are consistently seen in *Dll1* *^Dll4ki/+^* skeletons.

In summary, our *cis*-inhibition assays (Fig 5E) reveal a functional difference between DLL1 and DLL4 that was not evident in the *trans*-activation assays (Fig 5D and 5F): DLL4, but not DLL1, is a potent *cis*-inhibitor of NOTCH1 and *cis*-inhibition by DLL4 can significantly reduce Notch activation. Our *in vitro* results are consistent with our *in vivo* data: they can explain both why DLL4 appears to be a weaker activator of Notch signalling than DLL1 during somitogenesis in our transgenic mice and why transgenic DLL4 has a dominant effect on segmentation in *Dll1* ^*Dll4ki/+*^ mice (see Discussion and Fig 6). We propose that in the PSM, DLL1 is a more efficient net activator of Notch than (ectopic) DLL4 because it does not efficiently *cis*-inhibit Notch.

### The *cis*-inhibitory potential of DLL4 is mediated by its extracellular domain

In order to identify the protein domain that mediates *cis*-inhibition by DLL4, we cloned chimeric *Dll1* and *Dll4* ORFs by swapping extracellular domains (resulting in DLL4-ICD+TM/DLL1-ECD, termed DLL4-DLL1ECD, or DLL1-ICD+TM/DLL4-ECD, termed DLL1-DLL4ECD; ICD, intracellular domain; TM, transmembrane domain; ECD, extracellular domain; Fig 5G top). We introduced the chimeric *Dll1-4* ORFs transiently into HeLa-N1 cells and performed co-culture assays analogous to the *cis*-inhibition experiments with non-chimeric DLL1 and DLL4 shown in Fig 5E (Fig 5G; S9 Fig). Measurement of Notch activity showed similarity between DLL1 and DLL4-DLL1ECD as well as between DLL4 and DLL1-DLL4ECD (Fig 5Ga’, 5Gb’ and 5Gc’; n = 6; compare with Fig 5Ea’, 5Eb’ and 5Ec’). Particularly the statistically significant differences between bars in Fig 5Gb’ clearly indicate that DLL4-DLL1ECD enhances, but DLL1-DLL4ECD reduces Notch activation by DLL1. As both chimeric ligands localise to the cell surface (S9B and S9C Fig; S6 Table) and are able to *trans*-activate Notch in a range similar to DLL1 and DLL4 (S9D Fig) these results show that *cis*-inhibition is mediated by the extracellular domain of DLL4. This observation is consistent with studies that showed that the DSL domain as well as EGF repeats 4–6 of Serrate are essential for *cis*-inhibition in *Drosophila* although these EGF repeats are not well conserved between Serrate and Delta ligands [24,65,66]. Analysis of Notch activation by chimeric proteins in which smaller domains of the extracellular regions are swapped will help to precisely map the *cis*-inhibitory domain in DLL4.

## Discussion

The presence of several Notch receptors and ligands in mammals offers a multitude of possible receptor-ligand interactions; whether different combinations of receptor and ligand qualitatively or quantitatively vary in their signalling output is largely unknown. In this study, we focus on the mouse Notch ligands DLL1 and DLL4 and find functional differences *in vivo*, which are particularly apparent in the PSM: DLL4 cannot replace DLL1 during axial segmentation, and a striking dominant segmentation phenotype in *Dll1* ^*Dll4ki/+*^ mice hints towards an inhibitory function of ectopically expressed DLL4 in the PSM. We examined the possibility that differential *cis*-inhibition contributes to the phenotype and our *in vitro* Notch activation data are indeed consistent with this possibility (Fig 6), but do not exclude that other factors may contribute (see below).

### Direct comparison of DLL1 and DLL4 equally expressed in embryos uncover context-dependent differences in their ability to activate Notch

Mesodermal expression of DLL1 and DLL4 from the *Hprt* locus on a *Dll1* mutant background caused different phenotypes providing first hints that DLL1 and DLL4 are functionally different during early embryogenesis: CAG:DLL1 largely rescued the somitogenesis defects (Fig 1Eb and 1Ec) indicating that expression from this heterologous locus is strong enough to rescue the *Dll1* null segmentation phenotype. In contrast, CAG:DLL4 expressed from the same locus failed to sufficiently activate Notch (Fig 1Ee–1Eg). Our *Dll1* ^*Dll4ki*^ knock-in data independently confirm and extend the *CAG*:*Dll4* expression data, corroborating the inability of DLL4 to substitute for DLL1 function in the PSM (compare Figs 1Ee, 1Ef and 3Ae). We also show that the level of redundancy depends on the developmental process: there is essentially no redundancy during segmentation (Fig 3Ac, 3Ae, 3Bc and 3Be), partial redundancy in myoblast differentiation (Fig 3C–3F) and full redundancy in retinal progenitor maintenance (Fig 4). The effects on myogenesis and retinal development confirm that functional DLL4 is expressed from the *Dll1* ^*Dll4ki*^ allele (Figs 3De, 3Ee, 3Fe and 4B–4D).

Different protein levels of DLL1 and DLL4 are unlikely to account for the different phenotypes observed. Both proteins are expressed from identical genomic sites and the comparison of levels of bicistronic GFP (Fig 1C), transcripts (Fig 2E) and HA-tagged proteins (Fig 1D) confirm similar expression levels. Consistently, we find similar steady state levels, surface representation and half-lives of both ligands in CHO cells (Fig 5B and 5C; S5D–S5F Fig). Furthermore, immunohistochemistry using anti-DLL4 antibodies show similar levels of endogenous DLL4 and *Dll1*-driven DLL4ki expression in the retina (Fig 4E) as well as similar localisation of ectopic DLL4 and endogenous DLL1 at the cell surface within the PSM (Fig 2F). In our mouse models, we expressed untagged *Dll1* and *Dll4* transgenes to avoid alteration of protein function by the tag. As a consequence, we were unable to directly compare DLL1 and DLL4 levels *in vivo* and therefore cannot exclude small differences that may have contributed in part to the observed phenotype; strong differences are not indicated in the controls mentioned above.

Also, it is very unlikely that DLL1 or DLL4 have functions other than interacting with and activating Notch receptors. Although it has been previously suggested that the intracellular domain of DLL1 may influence gene transcription in the signal sending cell [67,68], we were unable to reproduce these *in vitro* results and showed that overexpression of the intracellular domain of DLL1 does not cause a phenotype in mice [42]. Collectively, the distinct ability to *cis*-inhibit Notch is a plausible explanation for the context-dependent DLL1-DLL4-divergence.

### Distinct *cis*-inhibitory capacity of DLL1 and DLL4 *in vitro*

The ability of vertebrate DLL homologues to *cis*-inhibit Notch has been suggested before: overexpression of truncated DLL1 proteins lacking the intracellular domain in *Xenopus*, chicken and mouse embryos show dominant-negative effects on Notch signalling that are likely to be caused by *cis*-inhibition of Notch [53,69,70]. In primary human keratinocyte cultures, expression of DLL1 (and truncated DLL1^T^) renders cells unresponsive to Delta signals from neighbouring cells and controls differentiation of stem cells [71]. Our data show for the first time that DLL4 is a strong *cis*-inhibitor of Notch signalling, far stronger than DLL1. We have examined *cis*-inhibition in various types of cultures, in NOTCH- and DLL-expressing HeLa cells with and without co-culture of empty or DLL-expressing CHO cells and with chimeric DLL1-4 proteins (Fig 5E and 5G). Furthermore, we have tested *cis*-inhibition of DLL1 and DLL4 ligands by NOTCH1 (Fig 5F). All those assays consistently show a strong reduction of Notch signalling by DLL4 when coexpressed with NOTCH1.

In our assays, DLL1 had no obvious *cis*-inhibitory effect (Fig 5Eb’; n = 6), which differs from earlier reports showing that vertebrate DLL1 proteins can *cis*-inhibit NOTCH1 [20,72–74]. This is likely due to different assay conditions: in these previous studies, DLL1 was derived from different vertebrate species or differently tagged, or different cell systems or higher ligand concentrations were used. In studies in which *cis*-inhibition of Notch by Delta and Serrate was compared, Delta displayed a relatively weaker *cis*-inhibitory potential [75,76].

The ability for strong *cis*-inhibition resides in the extracellular domain of DLL4 (Fig 5G) that physically interacts with the Notch extracellular domain. Possible causes for the higher *cis*-inhibitory potency of DLL4 as compared to DLL1 include a potentially higher Notch *cis*-binding affinity of DLL4 as determined for the *trans*-interaction *in vitro* [34] or different glycosylation patterns in the extracellular domains of DLL1 and DLL4 (DLL4 contains an additional O-fucosylation site in EGF5 and four additional N-glycosylation sites, three of which reside in the N-terminal domain, which is essential for Notch activation; e.g. [19]; sites predicted by www.cbs.dtu.dk/services/NetOGlyc).

### *cis*-inhibition by DLL4 *in vivo*

Our *in vitro* findings provide a possible explanation why DLL1 supports regular somite formation whereas DLL4 with its reduced net Notch activation potential is unable to do so. Heterozygous *Dll1* ^*Dll4ki/+*^ mice consistently exhibit kinky tails and irregular vertebrae (Figs 2B and 3Bd; S3 Fig) despite the presence of one wildtype *Dll1* allele, which should be able to support regular somitogenesis [53]. This finding strongly supports an *in vivo* inhibitory effect of DLL4 in the PSM, in which *Dll4* is ectopically expressed at physiological levels (similar to the endogenous *Dll1* levels; Fig 2E). Skeletal malformations observed in *Dll1* ^*Dll4ki/+*^ mice are distinct from phenotypes observed upon mild overexpression of *Dll1* in the paraxial mesoderm that include fused or split vertebral bodies and reduction of costal heads of ribs [77]. This supports the view that *cis*-inhibitory DLL4 acts in a dominant-negative manner partially overruling Notch activation by wildtype DLL1 causing axial skeleton defects in *Dll1* ^*Dll4ki/+*^ mice, similar to the effect of a truncated dominant-negative form of DLL1 expressed in the paraxial mesoderm [53]. An alternative explanation for the dominant segmentation effect in heterozygous *Dll1* ^*Dll4ki/+*^ mice could be a competition between DLL4 and DLL1 for NOTCH binding sites with DLL4 binding NOTCH more efficiently but activating it less efficiently than DLL1; although DLL1 has not been shown to be a more potent activator of NOTCH *in vitro* (Fig 5D; S6A, S6D and S6G Fig; [33,34]) we cannot exclude that this is the case in certain cellular contexts.

*cis*-Inhibition has been demonstrated to play a physiological role during fly development at the dorso-ventral border of the wing imaginal disc [15,16] and in photoreceptor precursors of the eye [17]. In vertebrates, the occurrence of *cis*-inhibition under physiological conditions is less clear but probable (see previous section). We did not observe apparent phenotypes in *Dll1* ^*Dll4ki/+*^ mice that indicate dominant-negative effects of DLL4 outside the PSM. However, we hypothesise that *cis*-inhibition may occur in the foetal arterial endothelium, where DLL1, DLL4 and NOTCH1 are coexpressed and where loss of DLL1 abolishes NOTCH1 activation [29], possibly due to *cis*-inhibition by DLL4.

The PSM is particularly well suited to test the functionality of Notch ligands *in vivo* because DLL1 is the only activating ligand endogenously expressed in this tissue [78] and *Dll4* mutants have no somitogenesis phenotype [39,40], so the analysis of Notch signalling is not complicated by the presence of several activators or confounded by composite phenotypes. However, two receptors, NOTCH1 and NOTCH2, are expressed in the PSM and may differ in their response to DLL1 or DLL4 binding.

The situation in myoblasts and other tissues is less clear. Outside the PSM, receptor and ligand expression typically exclude each other so that *cis*-inhibition can occur only during the short process in which the fate as receptor- or ligand-expressing cells is established [76,79,80]. That way, *cis*-inhibition may also be responsible for differences between DLL1 and DLL4 observed during myogenesis (Fig 3D–3F). Other reasons may contribute to or cause these differences: Firstly, further Notch receptors and ligands are expressed during myogenesis [81]. The contribution of individual Notch receptors to myogenesis is unknown but their function could vary [82]. Also, different ligands, including DLL1 and DLL4, have been shown to activate different Notch targets depending on the cell type *in vitro* [83,84]. A future thorough analysis of the functional divergence between DLL1 and DLL4 in myoblasts should aim at identifying the involved receptors and modulators (perhaps by *in vitro* analyses of myogenic or mesodermal progenitor cells including knock-down of individual factors) in order to understand the mechanisms underlying the observed phenotype.

Secondly, other processes may cause the divergence, e.g. modification of the ligands or receptors by glycosylation (may also play a role in the PSM). Activation of Notch by its ligands can be modulated by Fringe proteins. While glycosylation of Notch by LFNG enhances interaction with DLL1 in C2C12 cells [85] and with DLL4 in T cells *in vitro* [86], it appears to attenuate Notch signalling in the PSM [64,87]. However, we did not observe any shortcomings of DLL4 in the ability to *trans*-activate NOTCH1 compared to DLL1 when LFNG was present in the receptor-presenting cell (S6A–S6C Fig). The *trans*-activation potential of DLL1 and DLL4 could vary under certain conditions *in vivo*, perhaps depending on the glycosylation status, although our *in vitro* assays did not reveal any difference. Finally, the different extent of the functional difference between DLL1 and DLL4 observed in the PSM and during myogenesis may reflect the fact that mild changes of DLL1 activity affect the delicate Notch signalling in the PSM more readily than outside the PSM because somite patterning appears to be particularly sensitive to reduced Notch activity [88].

In conclusion, our genetic studies revealed a context-dependent functional divergence of the NOTCH ligands DLL1 and DLL4 in mice and provide a basis for a more extensive mechanistic analysis of this divergence in future studies. These will identify the relevant protein domain(s) and biochemical parameters and contribute to our understanding how different combinations of receptors and ligands determine the outcome of Notch signalling.

## Materials and Methods

### Cloning of constructs

#### *CAG*:*Dll1* and *CAG*:*Dll4 Hprt* targeting constructs

Untagged *Dll1* and *Dll4* ORFs were PCR-amplified with primer pairs Dll1-for (SpeI) ACT AGT GCC ACC ATG TCT TAC GGT CAA GGG TCC AGC / Dll1-rev (AseI) GAT CAT TAA TTC ACA CCT CAG TCG CTA TAA CAC ACT CAT CCT TTT C and Dll4-for (NheI) GCT AGC AAT TCA TGA CGC CTG CGT CCC G / Dll4-rev (NdeI) CAT ATG TTA TAC CTC TGT GGC AAT CAC. Restriction sites introduced via primers were used to insert PCR products into NheI-NdeI sites of a shuttle vector containing *IRES-GFP*. *Dll-IRES-GFP* constructs were then subcloned into pMP8.CAG-Stop using restriction enzymes SwaI and MluI. HA-tagged versions of the above constructs for quantification of protein levels (Fig 1D) were cloned in a similar way: PCR primers for *Dll1-HA* were Dll1-for (SpeI; see above) / Dll1-HA-rev (AseI) ATT AAT CTA AGC GTA ATC TGG AAC ATC GTA TGG GTA CAT ACT AGA CAC CTC AGT CGC TAT AAC ACA C. A C-terminal HA-tag was introduced to the *Dll4* ORF via gene synthesis (Life technologies) of the flanking regions of *Dll4-HA* lacking the central AflII/EcoRV *Dll4* fragment that was cloned into the synthesised fragment; the complete *Dll4-HA* was cloned into NheI/NdeI of the shuttle vector. HA-tagged constructs were Cre-recombined in bacteria of the recombination strain SW106 (NCI at Frederick).

#### *Dll4ki* targeting construct

The *Dll1* mini gene in the *Dll1* ^*Dll1ki*^ (*Dll1* ^*tm2Gos*^ *;* Fig 2A bottom; [37]) targeting vector was replaced with a *Dll4* mini gene by a 3-point ligation of a NsiI/KpnI fragment of the *Dll1ki* targeting construct (containing DT, the 5‘ homology region and the 5‘UTR of *Dll1* exon1), a NsiI/SmaI fragment of the same *Dll1ki* targeting vector (containing the vector backbone with *amp* ^*r*^, 3‘ homology region and floxed PGK-*neo* ^*r*^) and a KpnI/PmeI fragment (containing the complete *Dll4* ORF with start codon ATG followed by 3 Stop codons and—as an inactive remainder of a precursor clone—the genomic *Dll1* region from the last 42 bp of exon 9 to exon 11; gene structure resembled a *Dll3ki* targeting vector, [21]). To adjust the gene structure of the *Dll4ki* targeting vector to that of control *Dll1ki*, a 1,466 bp MfeI/BsiWI fragment (comprising last 134 bp of *Dll4* exon 9, the genomic *Dll1* remainder from exon 9–11 and 154 bp downstream) was replaced with a synthesised 1,293 bp MfeI/BsiWI DNA fragment (comprising *Dll4* exon 9 from the MfeI site, *Dll1* intron 9, *Dll4* exon 10, *Dll1* intron 10, *Dll1* exon 11 and 154 bp downstream; Life technologies); the only remaining difference between *Dll4ki* and control *Dll1ki* targeting vectors were the coding regions of *Dll4* or *Dll1* mini gene (ORFs from exon 1 to exon 9 and separate exon 10; exon 11 contains only one coding amino acid, i.e. a conserved valine).

#### CMV-Dll4mini-pA

The *Dll4ki* mini gene was released from the *Dll4ki* targeting construct with SacI/XbaI and cloned into expression vector pTracer-CMV (Invitrogen). *Dll1* and *Dll4* ORFs (Flag-tagged) were cloned into the multiple cloning site of pTracer-CMV and used as controls.

#### pHZ-attP-Jag1Myc

A DNA fragment containing *Jagged1-Myc* and flanking frt sites was synthesised (Life technologies) and inserted into the MluI site of the pHZ-attP vector [57].

#### *Dll1-attB*, *Dll4-attB* and chimeric *Dll1-4-attB*

The eGFP gene was removed from pNC-attB vector [57] by AfeI/HindIII digest, blunting ends with T4 DNA-Polymerase (Roche) and religation; Flag-tagged *Dll1* and *Dll4* ORFs were released from pTracer-CMV and inserted into pNC-attB-deltaGFP using restriction enzymes EcoRI/BamHI. To generate an alternative construct with HA-tagged DLL4, the HA-tag was added to the *Dll4* ORF by PCR with primer pair Dll4.up (EcoRI) GAA TTC ACC ATG ACG CCT GCG TCC CGG AGC G / Dll4.lowHA (NotI) GCG GCC GCT TAT TAT TAA GCG TAG TCT GGA ACG TCG TAT GGG TAT ACC TCT GTG GCA ATC ACA CAC TCG. *Dll4-HA* was inserted into EcoRI/NotI sites of pTracer-CMV and subcloned with PmeI/XbaI into AfeI/XbaI sites of pNC-attB-deltaGFP. Chimeric *Dll1-4* constructs were partly generated by gene syntheses (Life technologies) and cloned into pNC-attBdeltaGFP. For *Dll4-Dll1ECD* an NdeI-EcoRV fragment containing a part of *Dll1* ECD and complete *Dll4* TM and ICD synthesised; a BglII/NdeI *Dll1* ECD fragment was inserted into the gene synthesis vector and the whole chimeric ORF was inserted into pNC-attBdeltaGFP-Dll4 as an EcoRI/EcoRV fragment. For *Dll1-Dll4ECD* a BspEI/MfeI fragment containing part of *Dll4* ECD, *Dll1* ICD and part of *Dll1* ICD was synthesised. *Dll1* ICD was inserted as a MfeI/XbaI fragment and *Dll4* ECD as a ScaI/BspEI fragment; the chimeric ORF was then inserted into pNC-attBdeltaGFP-Dll4 as a ScaI/XbaI fragment. pNC-attBdeltaGFP with chimeric *Dll1-4* were used as transiently transfected expression vectors (CMV promoter).

### Generation and husbandry of transgenic mice

#### Ethics statement

All animal experiments were performed according to the German rules and regulations (Tierschutzgesetz) and approved by the ethics committee of Lower Saxony for care and use of laboratory animals LAVES (Niedersächsisches Landesamt für Verbraucherschutz und Lebensmittelsicherheit). Mice were housed in the central animal facility of Hannover Medical School (ZTL) and were maintained as approved by the responsible Veterinary Officer of the City of Hannover. Animal welfare was supervised and approved by the Institutional Animal Welfare Officer (Tierschutzbeauftragter).

#### Mouse strains

Wildtype (CD1 and 129Sv/CD1 hybrids), *Dll1* ^*loxP/loxP*^ [32], *Zp3*:*Cre* [45], *T(s)*:*Cre* [46], *Chx10*:*Cre* [89].

#### Generation of transgenic mice

Linearised targeting constructs were electroporated into E14TG2a (used for targeting of the *Hprt* locus; carry a deficient *Hprt* locus enabling HAT selection [90]) or 129Sv/cast (to target *Dll1*) mouse embryonic stem cells. Correct homologous recombinant clones were identified by long range PCR (Expand High Fidelity PCR System, Roche; primer sets for *Hprt* locus, HPRT-5‘typ.for2 ACC TGT TAG AAA AAA AGA AAC TAT GAA GAA CT / HPRT-CAG.rev1 GGC TAT GAA CTA ATG ACC CCG TAA TTG ATT ACT ATT A; for *Dll1* locus, EGF3’#1 TGT CAC GTC CTG CAC GAC G / EGF3’#2 GGT ATC GGA TGC ACT CAT CGC) and Southern blot analysis (5’ probe was a 316 bp BamHI/AvaII fragment 3.8 kb upstream of *Dll1* exon 1; 3’ probe was a 528 bp PCR fragment, CCT GTG AGA CTT TCT ACG TTG CTC / CAC AAC CAT GTC ACC TTC TAG ATT C, in *Dll1* intron 5) and used to generate chimeric mice.The *neo* ^*r*^ cassette was removed in the female germ line using *ZP3*:*Cre* mice. For embryo collection, mice were sacrificed by cervical dislocation, for adult skeletal preparations, mice were asphyxiated with CO_2_.

#### Genotyping

Genomic DNA was isolated from ear or tail biopsies or yolk sacs of embryos and used as template in PCRs with the following primer pairs: *CAG*:*Dll1* GAC GCT GAG GGG TAT GTG ATG / CTT GAG GCA TAC GCG AAA GAA GGT C; *CAG*:*Dll4* GAC GCT GAG GGG TAT GTG ATG / GCT CGT CTG TTC GCC AAA T; Cre-recombined *CAG*:*Dll1* or *-4* CGA GGT GAA GTT CGA GGG CGA C / GGG CAA CAG AGA AAT ATC CTG TCT C; gender (Y-chromosome PCR) CTG GAG CTC TAC AGT GAT GA / CAG TTA CCA ATC AAC ACA TCA C; *Cre* GCC TGC ATT ACC GGT CGA TGC AAC GA / GTG GCA GAT GGC GCG GCA ACA CCA TT; *Dll1* ^*wt*^ ACT CAC CTC TCC GTG GAC TGA AAG C / GGA GCT CCA GAC CTG CGC GGG; unrecombined *Dll1* ^*loxP*^ GCA TTT CTC ACA CAC CTC / GAG AGT ACT TGA TGG AGC AAG; Cre-recombined *Dll1* ^*loxP*^ CAC ACC TCC TAC TTA CCT GA / TGA AGT GTG GAC CCA TCA TC; *Dll1* ^*Dll4ki*^ AAG GAC AAC CTA ATC CCT GCC G / TGC CAC ATC GCT TCC ATC TTA C; *Dll1* ^*Dll1ki*^ CCT GGT TTC CGT GGA GCA TGG ACA / GGG TGC AGG AAG AGG AGA GGG CAG; *Dll1* ^*lacZ*^ ACT CCT GGG TCT TTG AAG AAG / TGT GAG CGA GTA ACA ACC CGT CGG ATT.

### Analyses of gene expression patterns and phenotypes

#### Whole mount *in situ* hybridisation

Embryos were collected in ice cold phosphate buffered saline (PBS) and fixed over night in 4% formaldehyde/PBS at 4°C. Hybridisation was performed following standard procedures [91] with digoxigenin labelled cDNA probes comprising the whole ORFs of *Dll1* or *Dll4*, *Dll1* exon 11, ~1kb of the 5‘ region of *Myogenin* cDNA [92] and *Uncx4*.*1* [47]. Photos were taken with the Leica Z6 APO microscope and Leica FireCam software.

#### Antibody staining

E15.5 embryos were fixed over night in 4% formaldehyde/PBS at 4°C, dehydrated in methanol and 2-propanol, embedded in paraffin and sectioned sagittally (10 μm). Antigene unmasking was performed by cooking sections 20 min in 10 mM Tris pH9.5/1 mM EDTA. Sections were blocked in 1% BSA/2% goat serum 1h at room temperature, incubated with a monoclonal mouse anti-myosin antibody (skeletal, fast, My32; dilution 1:250; Sigma-Aldrich) in blocking solution over night at 4°C, incubated with secondary goat biotinylated anti-mouse antibody (BA 9200; 1:200; Vector Laboratories) in blocking solution 1 hour at RT and stained with VECTASTAIN Elite ABC Kit and DAB (Vector Laboratories). Pictures were taken with a Leica microscope DM5000 B and Leica FireCam software.

#### Immunofluorescence

Embryos were fixed in 4% formaldehyde at 4°C, then cryoprotected in 30% sucrose and embedded in 7.5% gelatin:15% sucrose. Cryostat sections (12 μm) were used for immunofluorescence. Retinal sections were degelatinised at 37°C for 20 min, followed by treatment with 0.1 M glycine for 10 min at room temperature. Permeabilisation was performed using 0.5% TritonX-100 for 10 min. In the case of CHX10, P27 and ISL1 immunohistochemistry, antigen retrieval was performed after degelatinisation by boiling slides for 10 min in 0.01 M citrate buffer (pH 6). Primary antibodies used were anti-DLL4 (AF1389, R&D; 1:100), anti-N-Cadherin (610920, BD Transduction Lab; 1:100), anti-PHH3 (06–570, Upstate Biotech; 1:500), anti-ZO-1 (33–9100, Zymed; 1:50), anti-CHX10 (X1180P, Exalpha; 1:100), anti-P27 (PA5-27188, NeoMarkers; 1:100), anti-ISL1 (40.2D6, DSHB 1:100), anti-CRABP (Affinity Bioreagents 1:1,000). DAPI counterstain was used to visualise nuclei. Images were taken using a Leica DM5000 BA fluorescence microscope. A total of ~10,000 ISL1+ cells were counted in 4 control and 5 *Dll1* ^*Dll4ki*^ embryos and a total of ~2,500 CRABP+ cells were counted in 4 control and 4 *Dll1* ^*Dll4ki*^ embryos.

#### Whole mount immunofluorescence

Immunofluorescence staining of E9.5 old embryos was performed as described in [93]. Primary antibodies used were anti-DLL4 (AF1389, R&D; 1:50), anti-panCadherin (C1821, Sigma; 1:250) and anti-DLL1 (1F9 [21]; 1:50). Alexa-488/555 conjugated secondary antibodies (Invitrogen; 1:100) were used. Images were taken using OLYMPUS FV1000.

#### Venus fluorescence

Dissected E8.5 embryos in ice cold PBS were analysed under a Leica microscope DMI6000 B using LAS AF software.

#### Skeletal preparations

Skeletons of E18.5 mouse foetuses and adult mice were dissected and stained with Alcian blue and Alizarin red following standard procedures [53].

### Analyses of gene expression levels

#### Western blot analysis

Cells/embryos were lysed in 2x sample buffer (0.125 M Tris pH 6.8/4% SDS/20% glycin/5% beta-mercaptoethanol/0.025% bromphenol blue). Proteins were separated by SDS-PAGE and transferred to Immobilon-P Transfer membranes (Millipore) by wet tank blotting. Blots were blocked in 5% nonfat dried milk powder (AppliChem) in PBS/0.1% Tween 20. Primary antibodies: anti-HA HRP (rat monoclonal; clone 3F10, Roche; HRP, horseradish peroxidase-conjugated; 1:5,000–1:10,000), anti-Flag HRP (mouse monoclonal; clone M2, Sigma), anti-GFP HRP (mouse monoclonal; MACS molecular; 1:10,000), anti-DLL1 (1F9, rat monoclonal, [21] 1:1,000), anti-DLL4 (rabbit polyclonal against peptide C-GKIWRTDEQNDTLT; BioGenes; 1:50–1:100), anti-β-actin (mouse monoclonal; MP Biomedicals; 1:250,000–1:500,000), anti-β-tubulin I (Sigma; 1:500,000). Secondary antibodies: anti-mouse HRP, anti-rat HRP, anti-rabbit HRP (Amersham; 1:10,000). HRP was detected with ECL Western Blotting Detection Reagents (Amersham) with the Luminescent Image Analyser LAS-4000 (Fujifilm); signals were quantitated with ImageJ software.

#### Northern blot analysis

E11.5 mouse foetuses were collected in ice cold PBS and immediately frozen and kept at -80°C in lysis buffer (100 mM Tris pH7.5/500 mM LiCl/10 mM EDTA/1% LiDS (lithium dodecylsulphate)/5 mM DTT). polyA(+) RNA was isolated with magnetic OligodT beads (Novagene) following the manufacturer’s instructions. 2 μg of polyA(+) RNA was separated on a 1% agarose gel containing 6.6% formaldehyde in 1x MOPS buffer. RNA was transferred to a nylon membrane (Hybond-N+; Amersham) with 20x SSC, the membrane washed with 5x SSC and UV-crosslinked (Stratalinker, Stratagene). The blot was hybridised with radioactively labelled probes (*Dll1* 3‘ UTR, first 362 bp of *Dll1* exon 11 PCR-amplified with GTG TAA GAT GGA GC GAT GTG GCA / GGC AGT TGT GTT TCT AGT TCA AGG AAA G, RNA probe; actin, 1.2 kb SalI/SacI fragment of β-actin cDNA, DNA probe) over night at 65°C in Church buffer (300 mM NaPi pH6.7/5 mM EDTA/7% SDS). Signals were detected with a phospho imager (FLA-7000; Fujifilm) and quantified using ImageJ software.

#### Southern blot analysis

DNA was isolated from CHO^attP^ cells, digested with various restriction enzymes (*EcoR*I, *EcoR*V, *Hind*III, *Mfe*I, *Xba*I; NEB) over night and separated on a 0.7% agarose gel. Blotting, crosslinking, hybridisation (radioactively labelled DNA probe, i.e. a DraI/HindIII fragment of the pNZ-attP vector [57] containing the *attP* site and part of the *Hyg* ^*r*^ gene) and signal detection as for Northern blot analysis.

### Cell culture experiments

#### Culture and transfection

CHO and HeLa-N1 cells were cultured in DMEM/F12 (Invitrogen) cell culture medium with 10% FCS (Biochrom AG), 1% GlutaMAX (Gibco) and 1% Pen/Strep (Gibco). Transfection of cells was performed with PerFectin (Genlantis) following the manufacturer’s instructions. CHO cells transiently expressing the *Dll1* ^*Dll4ki*^ mini gene were transfected on 6-well dishes with 2 μg of DNA and lysed after 18 hours. CHO^attP-JAG1Myc^ cells were generated by electroporation of *pHZ-attP-Jag1Myc* plasmid into CHO cells and selection with 500 μg/ml Hygromycin B (S4 Fig). Integration of the *attP* site was checked by PCR using primers attP-for TAC TGA CGG ACA CAC CGA AGC / attP-rev GAA CGG CAC TGG TCA ACT TGG. CHO^attP-JAG1Myc^ cells were transfected with pCAGGs-Flpo [94] and *Jag1-Myc* was deleted by Flippase mediated recombination of the flanking FRT sites, thereby generating CHO^attP^ cells. After transfection with attB vectors [57], cells were selected for correct integration with 250 μg/ml Zeocin.

#### Biotinylation assay

Cells were plated on 6 cm dishes. At 80% confluence, the cells were washed twice with ice cold PBS supplemented with 0.1 mM CaCl_2_ and 1 mM MgCl_2_ (PBS-C/M). After incubation on ice for 10 min with PBS-C/M, cells were treated with Sulfo-NHS-LC (Pierce; 0.25mg/ml in PBS-C/M) for 40 min to bind all proteins present on the cell surface. To quench the biotin reaction, cells were washed twice with PBS-C/M and incubated with 100 mM glycine in DMEM on ice for 30 min. After washing with PBS, the cells were lysed in lysis buffer [50 mM Tris-HCl pH7.6, 150 mM NaCl, 1 mM EDTA, 1% TritonX-100, 0.25% DOC (Sodium-desoxycholate), 0.1% SDS] supplemented with Complete Protease Inhibitor Cocktail Tablets (Roche) on ice for 30 min. Next, the samples were sonified and centrifuged at 10,000 x g for 15 min at 4°C to remove cell debris. Using NeutrAvidin beads (Thermo Scientific) pre-washed in lysis buffer, biotinylated proteins were immunoprecipitated and analysed [95]. Protein amounts were calculated as follows: total DLL protein in lysate = signal intensity of input x total volume of lysate/loaded volume; DLL protein on surface = signal intensity of IP x total volume of IP eluate/loaded volume; relative cell surface levels = DLL protein on surface / total DLL protein in lysate.

#### *trans*-activation assay

For analysis of Notch *trans*-activation, HeLa-N1 cells were transfected with 2 μg of RBP4-Luciferase reporter and 0.5 μg of firefly renilla-Luciferase on 6-well dishes using PerFectin (Genlantis). To analyse the effect of NOTCH1 glycosylation, HeLa-N1 cells were transiently transfected with 1.5 μg of *attB-LfngHA*. For analysis of *cis*-inhibition, HeLa-N1 cells were transiently transfected with 200 ng of Flag-tagged *attB-Dll* constructs. For analysis of the *cis*-inhibitory potential of NOTCH1, the ligand expressing CHO^attP-DLL^ cells were transfected with 6 μg of Flag-tagged NOTCH1ΔC (NOTCH1 lacking the C-terminal 56 amino acids) expression vector. Expression of proteins was validated by Western blot analyses. Co-cultivation of HeLa-N1 and CHO^attP^ cell lines was performed with 1.25 x10^5^ cells each on 12-well dishes for 24 hours; pure HeLa-N1 cultures with 2.5 x10^5^ cells on 12-well dishes for 48 hours. N-linked glycosylation was blocked by cultivating the cells in medium containing 1 μg/ml tunicamycin for 22 hours. For Luciferase detection the Dual-Luciferase Reporter Assay System (Promega) was used according to the manufacturer’s instructions; probes were analysed in a TurnerBioSystems luminometer with Glomax software. Each (co-)culture was performed in duplicates and every lysate was measured twice; the mean of the four measurements counted as n = 1.

#### Immunofluorescence

Cells were fixed in 4% formaldehyde for 10 minutes on ice, permeabilised with 1% Triton-X100 for 15 minutes at RT and washed in PBS for 30 minutes. Cells were then incubated for 5 minutes in 0.2% glycine, washed for 15 minutes with 0.1% Triton-X100 in PBS for 30 minutes, blocked with 5% FCS/0.1% Triton-X100/PBS for 1 hour at RT and incubated for 1 hour with primary antibodies (anti-Flag, clone M2, Sigma; 1:4,000), washed again with 0.1% Triton-X100 in PBS for 30 minutes and incubated with secondary antibodies (anti-mouse-Alexa488, Invitrogen; diluted in blocking solution 1:100) for 1 hour. After another washing step (0.1% Triton-X100 in PBS for 30 minutes) nuclei were stained by incubating the cells with TO-PRO3 (Invitrogen; diluted 1:1,000 in PBS) for 30 minutes at RT. After washing in PBS and water cells were mounted in ProLong-Gold antifade reagent (Life Technologies) and analysed with a Leica DM IRB microscope with a TCS SP2 AOBS scanhead.

#### Determination of half-lives of DLL1-Flag and DLL4-Flag

Cells were cultured in medium containing 100 μg/ml Cycloheximide and were lysed in 2x sample buffer (0.125 M Tris pH 6.8/4% SDS/20% glycin/5% beta-mercaptoethanol/0.025% bromphenol blue) after 30 minutes, 1 hour, 2 hours, 3 hours, 4 hours, 6 hours, 8 hours, 10 hours, 12 hours, 14 hours, 16 hours, 18 hours, and 24 hours. Protein levels were analysed on Western blots and quantified with ImageJ software. Half-lives were determined using Prism software (GraphPad).

### Statistical analyses

Statistical analyses were performed using Prism software (GraphPad). Luciferase measurements were analysed by one-way ANOVA and activities obtained with each protein were compared using Bonferoni’s Multiple Comparison Test with a significance level of 0.05. Means for all three DLL1-and DLL4-HA clones in Fig 1D, cell counts in the retina and cell surface levels of chimeric ligands were analysed using the Student’s t-test.

## Supporting Information

S1 Fig Quantification of GFP, DLL1-HA and DLL4-HA expressed from the *Hprt* locus. **(A)** Exemplary blot for the analysis of GFP levels expressed in transgenic embryos (Fig 1B and 1C) by Western blot analysis of embryo lysates with anti-GFP antibodies and anti-β-actin (for normalisation) to compare levels of *CAG*:*DLL1-* and *CAG*:*DLL4-IRES-Venus* expression. **(B)** Direct comparison of DLL1-HA and DLL4-HA levels in three independent embryonic stem cell clones (ES cells, E14TG2a) with single copy integrations of *CAG*:*Dll1-HA* (clones A11, B2, B7) and *CAG*:*Dll4-HA* (clones A1, A10, B10) into the *Hprt* locus (Fig 1D) by Western blot analysis using anti-HA and anti-β-tubulin (for normalisation) antibodies. Three independent lysates of each clone (experiment 1–3) were analysed twice (WB1, WB2); ES cells, lysate of unelectroporated E14TG2a cells as negative control.
(TIF)

S2 Fig Correct DLL4 protein expression from the *Dll4* minigene. *In vitro* test of DLL4 protein expression from the *Dll4* mini gene (under the control of a CMV promoter) in comparison to *Dll4* cDNA and *Dll1* cDNA in transiently transfected CHO cells. Cell lysates were analysed with DLL4, DLL1 and anti-β-actin (loading control) antibodies on a Western blot. Identical signals obtained from the *Dll4* mini gene and *Dll4* cDNA at the size of ~100 kDa confirmed correct expression of DLL4 protein from the *Dll4* mini gene; anti-DLL4 and anti-DLL1 antibodies specifically recognised the correct DLL paralogue and gave no endogenous CHO signals in the negative control (untransfected CHO cells). As the cells were transfected transiently, this blot cannot be analysed quantitatively.
(TIF)

S3 Fig Defects in the axial skeletons of heterozygous *Dll1* ^*Dll4ki/+*^ adults. Skeletal preparations of seven adult *Dll1* ^*Dll4ki/+*^ males (1–7; 4 to 8 months old) are largely normal but consistently exhibit irregularities (arrows) in the rib cage (top) and/or tail (bottom) suggesting a mild dominant-negative effect of transgenic *Dll4* (see main text and Discussion).
(TIF)

S4 Fig Validation of unique *attP* site integration in CHO^attP^ cells. **(A)** Map of the genomic integration of the pHZ-attP construct containing *attP* site, *Hyg* ^*r*^, *Zeo* ^*r*^ and *frt*-flanked *Jagged1* (*Jag1*). The position of restriction sites and of the probe used for Southern blot analysis are indicated. The flanking genomic sequence and position of restriction sites outside the vector is unknown. **(B)** Southern blot analysis of DNA isolated from CHO^attP-JAG1^ cells shows a single product for each digest indicating a single genomic integration of the *attP* construct (the 3.3 kb *EcoR*I fragment is entirely derived from the integrated construct and served as a control). **(C)** ΦC31 integrase-mediated insertion of *Dll1* and *Dll4* into CHO^attP-JAG1^ generates CHO^attP-JAG1-DLL1^ or CHO^attP-JAG1-DLL4^ cells used in S6D Fig; JAG1 is Myc-tagged, DLL1 and DLL4 are Flag-tagged. **(D)** Excision of *Jag1* by FLP recombination results in CHO^attP^ cells that were subsequently used for the generation of CHO^attP-DLL1^ and CHO^attP-DLL4^ cells.
(TIF)

S5 Fig DLL1 and DLL4 stably expressed in CHO^attP^ cells: Protein levels, surface localisation and half-lives. **(A)** Exemplary Western blot used for the analysis of protein levels in Fig 5B. CHO^attP^ cells were used as negative control; β-actin was used for normalisation. **(B)** Extended Western blot analysis of protein levels including additional clones of CHO^attP-DLL1^ and CHO^attP-DLL4^. Expression levels varied to some degree, but DLL4 levels were not below DLL1 levels. Clone CHO^attP-DLL1^ C6 is the same in Fig 5B and can be used to compare values between both Figs. Error bars represent SEM; ns, not significant; *, P<0.05; **, P<0.01. **(C)** Exemplary Western blot used for the quantification of cell surface protein levels by biotinylation in Fig 5C. The protein amount was quantitated and the relative protein surface level was calculated as described in Materials and Methods. **(D)** Immunocytochemistry of fixed CHO^attP-DLL1^ and CHO^attP-DLL4^ cells. Flag-tagged ligands were visualised using anti-Flag antibodies. DLL1 and DLL4 are present at the cell surface. **(E,F)** Determination of DLL1 and DLL4 protein half-lives. (E) DLL1 and DLL4 half-lives analysed using two different clones for each cell line. (F) Average protein decay of the clones shown in (E): DLL4 is more stable (half-life 7.3 hours) than DLL1 (half-life 4.9 hours). Dashed lines indicate the 95% confidence interval.
(TIF)

S6 Fig Influence of the presence of LFNG or JAG1 or of inhibition of N-glycosylation on Notch activation. **(A)** Notch *trans*-activation assays with co-cultures of CHO^attP^ (negative control), CHO^attP-DLL1^ and CHO^attP-DLL4^ cells with Notch reporter expressing HeLa-N1 cells (cf. Fig 5D) without and with transient expression of LFNG-HA. Expression of LFNG in HeLa-N1 cells decreases the *trans*-activation ability of DLL4 to levels similar to DLL1, whose activation potential is slightly increased. **(B)** Scheme of interactions in co-cultivation assays with possible influence of LFNG expressed in HeLa-N1 cells. **(C)** Western blot showing the expression of LFNG-HA in HeLa-N1 cells used in (A); β-tubulin, loading control. **(D)** Notch *trans*-activation assays with CHO^attP^, CHO^attP-DLL1-Flag^ and CHO^attP-DLL4-HA^ cells without or with stable expression of JAG1-Myc (S4C and S4D Fig) in co-culture with Notch reporter expressing HeLa-N1 cells. Stable coexpression of JAG1 in DLL1 or DLL4 presenting cells does not significantly change Notch activation. DLL4 plus JAG1 activate the receptor more efficiently than DLL1 plus JAG1. **(E)** Scheme of different possible interactions in co-cultivation assays with or without stable JAG1 expression. **(F)** Western blot showing the expression of DLL1-Flag, DLL4-HA and JAG1-Myc used in (D). β-actin, loading control. **(G)** Notch *trans*-activation assay with co-cultures of CHO^attP^, CHO^attP-DLL1-Flag^ and CHO^attP-DLL4-Flag^ cells with Notch reporter expressing HeLa-N1 cells in the absence or presence of tunicamycin, an inhibitor of N-linked glycosylation. Cultivation in the presence of 1 μg/ml tunicamycin (dissolved in DMSO) decreases the activating potential of both DLL1 and DLL4 to similar levels. **(H)** Treatment with 1 μg/ml tunicamycin results in a size shift of DLL1 and DLL4 (asterisks) indicating an efficient block of N-glycosylation. DLL1 and DLL4 contain one and five N-glycosylation sites, respectively; consistently, the shift is stronger for DLL4. Error bars represent SEM; ns, not significant; *, P<0.05; **, P<0.01, ***,P<0.001.
(TIF)

S7 Fig DLL1 and DLL4 transiently expressed in HeLa-N1 cells: Interactions, expression and robustness of *cis*-inhibition assay. **(A)** Schema of all different cell interactions possible in co-cultivation assays with untransfected and transfected HeLa-N1 cells, CHO^attP^ cells and CHO^attP-DLL1^ cells shown in Fig 5Ea (left) and 5Eb (right). **(B,C)** Transient expression of ligands was tested by Western blot analysis with anti-Flag antibodies (DLL1 and DLL4 labelled with asterisks). As expected, transient expression levels vary; in some experiments (exp 1–3; B), DLL4 was expressed more strongly than DLL1, while in other experiments (exp 4–6; C) DLL1 was expressed more strongly than DLL4. Cells of all six transfections shown were used in Notch activation assays shown in Fig 5E (n = 6). Separate analyses of Notch activation assays performed with cells that express either **(D)** DLL4 (B; n = 3) or **(E)** DLL1 (C; n = 3) more strongly result in diagrams very similar to Fig 5E, demonstrating that Notch activation assays were robust and independent of transient expression levels; a’,b’,c’ in (D,E) refer to a,b,c in Fig 5E. Error bars represent SEM; ns, not significant; *, P<0.05; **, P<0.01; ***, P<0.001; ****, P<0.0001.
(TIF)

S8 Fig Expression of NOTCH1 in transiently transfected CHO^attP^ cells. Lysates of CHO^attP-DLL1^ or CHO^attP-DLL4^ cells transiently transfected with *Notch1-Flag* (*N1∆C*) were analysed on a Western blot with anti-Flag antibodies. All transfected cell populations (used in Fig 5F) express NOTCH1. CHO, negative control; β-actin, loading control.
(TIF)

S9 Fig Chimeric DLL1-DLL4 proteins transiently expressed in HeLa-N1 cells: Expression, surface localisation and *trans*-activation efficiency. **(A)** Expression of chimeric ligands in transiently transfected HeLa-N1 cells was checked by Western blot analysis with anti-Flag antibodies. Lysates of cells from all transfections (exp 1–6) used in Fig 5G are shown, DLL4-DLL1ECD and DLL4-DLL1ECD signals indicated with asterisks; HeLa-N1, untransfected negative control; Flag background signal or β-tubulin, loading control. **(B)** Immunofluorescence of CHO^attP^ cells stably expressing Flag-tagged chimeric ligands with anti-Flag antibodies (Alexa488-conjugated secondary antibody, green; nuclei stained with TO-PRO3, blue) shows cell surface localisation of both. **(C)** Surface biotinylation assays of chimeric proteins stably expressed in CHO^attP^ cells indicate higher cell surface levels of DLL4-DLL1ECD than of DLL1-DLL4ECD; *, P<0.05. **(D)** Notch *trans*-activation assays with CHO^attP^ cells, cells stably expressing non-chimeric proteins (CHO^attP-DLL1-Flag^, CHO^attP-DLL4-HA^) and cells expressing chimeric proteins (CHO^attP-DLL1-DLL4ECD-Flag^ and CHO^attP-DLL4-DLL1ECD-HA^ cells) co-cultured with Notch-reporter containing HeLa-N1 cells. The results show that all ligands, non-chimeric or chimeric, activate Notch; DLL4 and DLL4-DLL1ECD are slightly more efficient activators in this assay. Error bars represent SEM; ns, not significant; **, P<0.01.
(TIF)

S1 Table Raw data of GFP protein level analysis in Fig 1C. In two independent experiments six E8.5 embryos of each genotype were lysed in 2x sample buffer and analysed by Western blot using anti-GFP and anti-β-actin antibodies. The signals were quantified using ImageJ software. The GFP signals were divided by β-actin for normalisation of different amounts loaded; additionally, the values of each experiments were divided by the *CAG*:*Dll1* value for normalisation.
(PDF)

S2 Table Raw data of DLL1-HA and DLL4-HA protein level analysis in Fig 1D. In three independent experiments, confluent embryonic stem cells on 6 cm dishes were lysed in sample buffer and analysed on Western blots using anti-HA- and anti-β-tubulin antibodies. Three embryonic stem cell clones expressing DLL1-HA and DLL4-HA from the recombined *Hprt* locus were analysed; every lysate was loaded twice (#WB). HA and β-tubulin signals were quantified using ImageJ software. HA signals were divided by β-tubulin signals (normalisation of different amounts loaded) and the average value of every clone in every experiment was calculated and analysed using Prism software (GraphPad).
(PDF)

S3 Table Raw data of DLL1-Flag and DLL4-Flag protein level analysis in Fig 5B. Cells were plated on 6 cm dishes, cultured over night, lysed and analysed on Western blots with anti-Flag and anti-β-tubulin antibodies in four independent experiments. Each cell lysate was loaded twice (#WB). Two clones of each cell line were tested. Flag and β-actin signal intensities were determined using ImageJ. For normalisation, Flag signals were divided by ß-actin signals and the average for every clone in every experiment was calculated. Finally, all samples were normalized to clone CHO^attP-DLL1^ B5 to account for differences between the individual experiments.
(PDF)

S4 Table Raw data of DLL1-Flag and DLL4-Flag protein level analysis in S5B Fig. Protein levels of additional CHO^attP-DLL1^ and CHO^attP-DLL4^ clones were analysed as described in S3 Table.
(PDF)

S5 Table Raw data of DLL1-Flag and DLL4-Flag cell surface level analysis in Fig 5C. Lysates from biotinylation assays were analysed on Western blots and signals were quantified using ImageJ software. Each lysate was analysed at least twice (#WB) and the average was used as result from the respective experiment.
(PDF)

S6 Table Raw data of cell surface level analysis of chimeric DLL1-DLL4 proteins in S9C Fig. Biotinylation assays were performed to determine the relative cell surface levels of the chimeric proteins. The lysates were analysed on Western blots twice (#WB) and the average was calculated.
(PDF)
