# Supplementary figures and images for: Context-Dependent Functional Divergence of the Notch Ligands DLL1 and DLL4 In Vivo
Source: PLoS Genet. 2015 Jun 26;11(6):e1005328. doi: 10.1371/journal.pgen.1005328 (PMC4482573; doi:10.1371/journal.pgen.1005328)

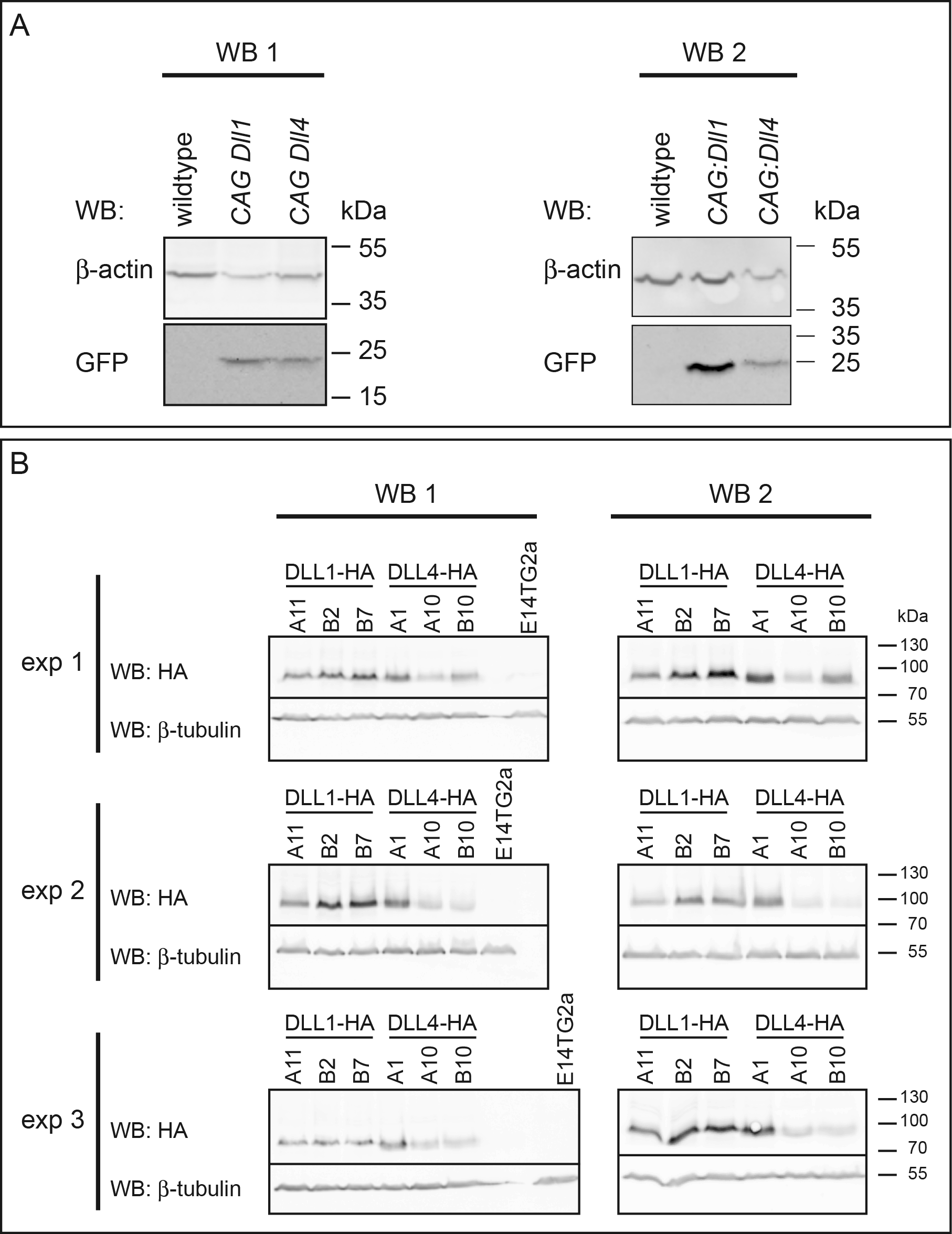

Supplement: S1 Fig — (A) Exemplary blot for the analysis of GFP levels expressed in transgenic embryos (Fig 1B and 1C) by Western blot analysis of embryo lysates with anti-GFP antibodies and anti-β-actin (for normalisation) to compare levels of CAG:DLL1- and CAG:DLL4-IRES-Venus expression. (B) Direct comparison of DLL1-HA and DLL4-HA levels in three independent embryonic stem cell clones (ES cells, E14TG2a) with single copy integrations of CAG:Dll1-HA (clones A11, B2, B7) and CAG:Dll4-HA (clones A1, A10, B10) into the Hprt locus (Fig 1D) by Western blot analysis using anti-HA and anti-β-tubulin (for normalisation) antibodies. Three independent lysates of each clone (experiment 1–3) were analysed twice (WB1, WB2); ES cells, lysate of unelectroporated E14TG2a cells as negative control. (TIF) [file pgen.1005328.s001.tif]

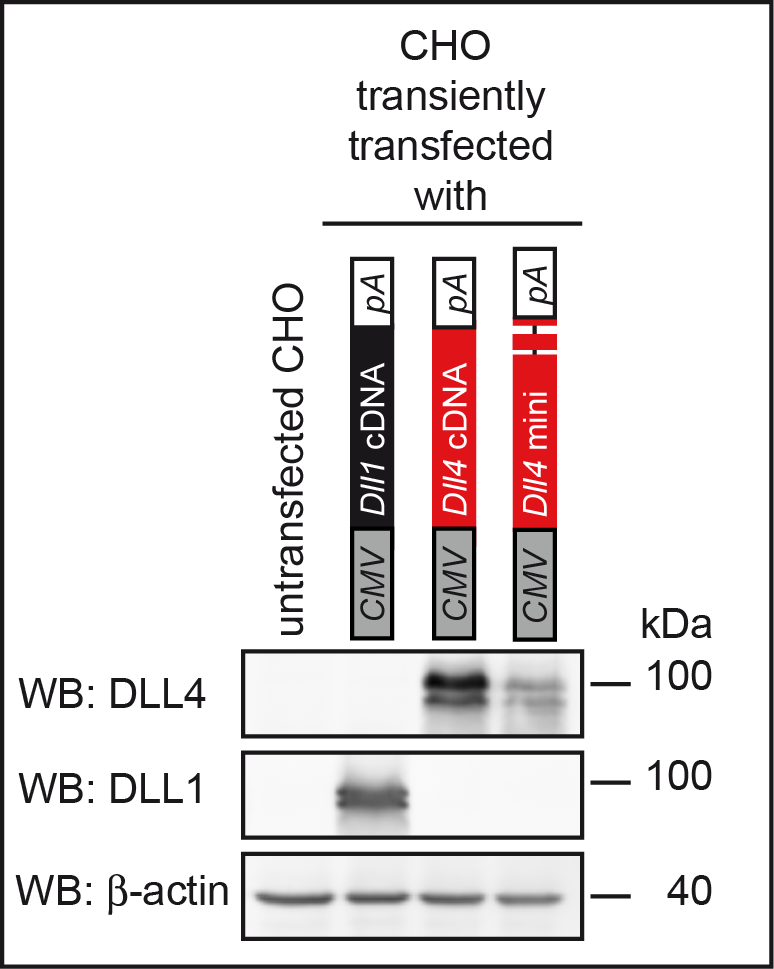

Supplement: S2 Fig — In vitro test of DLL4 protein expression from the Dll4 mini gene (under the control of a CMV promoter) in comparison to Dll4 cDNA and Dll1 cDNA in transiently transfected CHO cells. Cell lysates were analysed with DLL4, DLL1 and anti-β-actin (loading control) antibodies on a Western blot. Identical signals obtained from the Dll4 mini gene and Dll4 cDNA at the size of ~100 kDa confirmed correct expression of DLL4 protein from the Dll4 mini gene; anti-DLL4 and anti-DLL1 antibodies specifically recognised the correct DLL paralogue and gave no endogenous CHO signals in the negative control (untransfected CHO cells). As the cells were transfected transiently, this blot cannot be analysed quantitatively. (TIF) [file pgen.1005328.s002.tif]

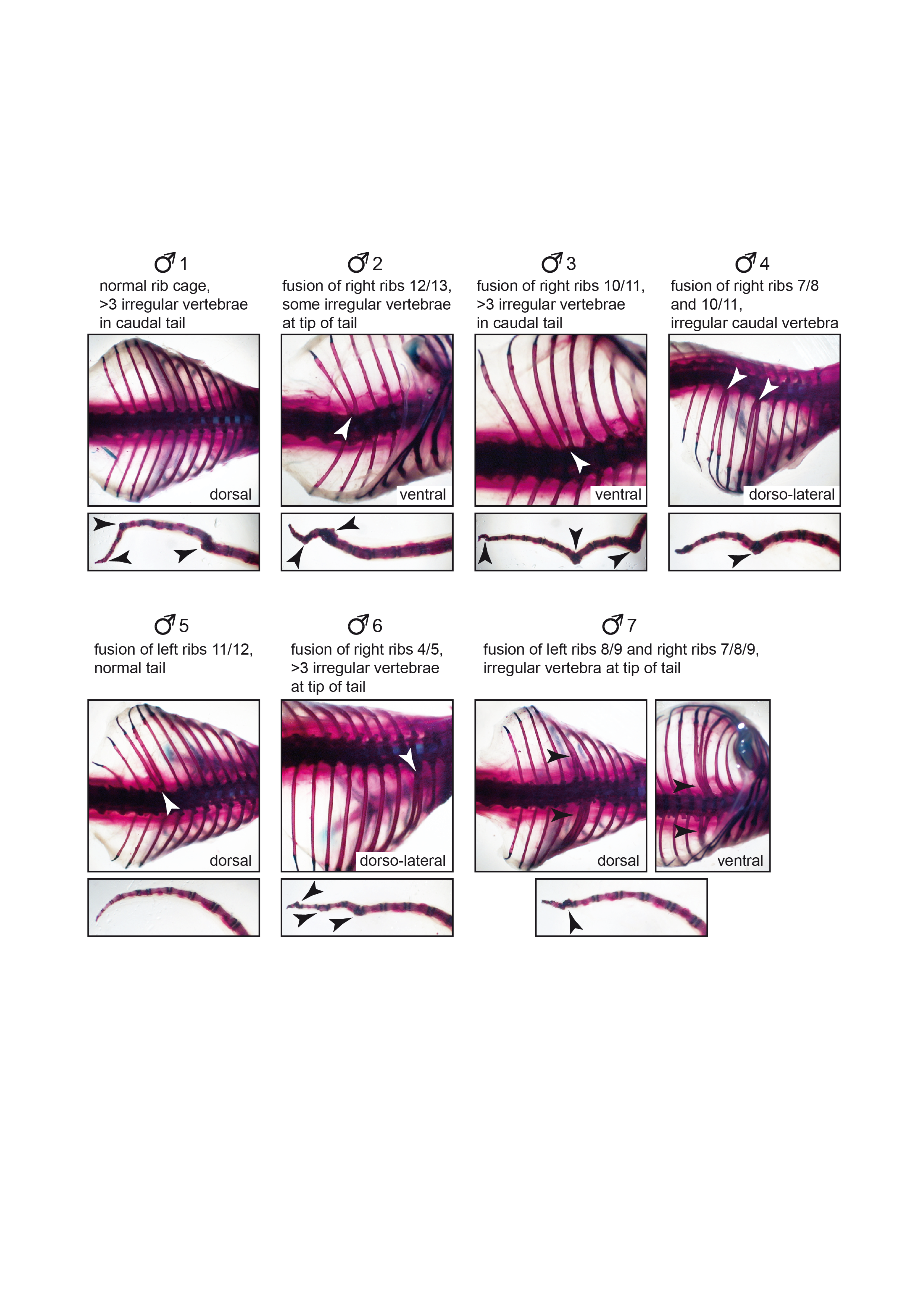

Supplement: S3 Fig — Skeletal preparations of seven adult Dll1 Dll4ki/+ males (1–7; 4 to 8 months old) are largely normal but consistently exhibit irregularities (arrows) in the rib cage (top) and/or tail (bottom) suggesting a mild dominant-negative effect of transgenic Dll4 (see main text and Discussion). (TIF) [file pgen.1005328.s003.tif]

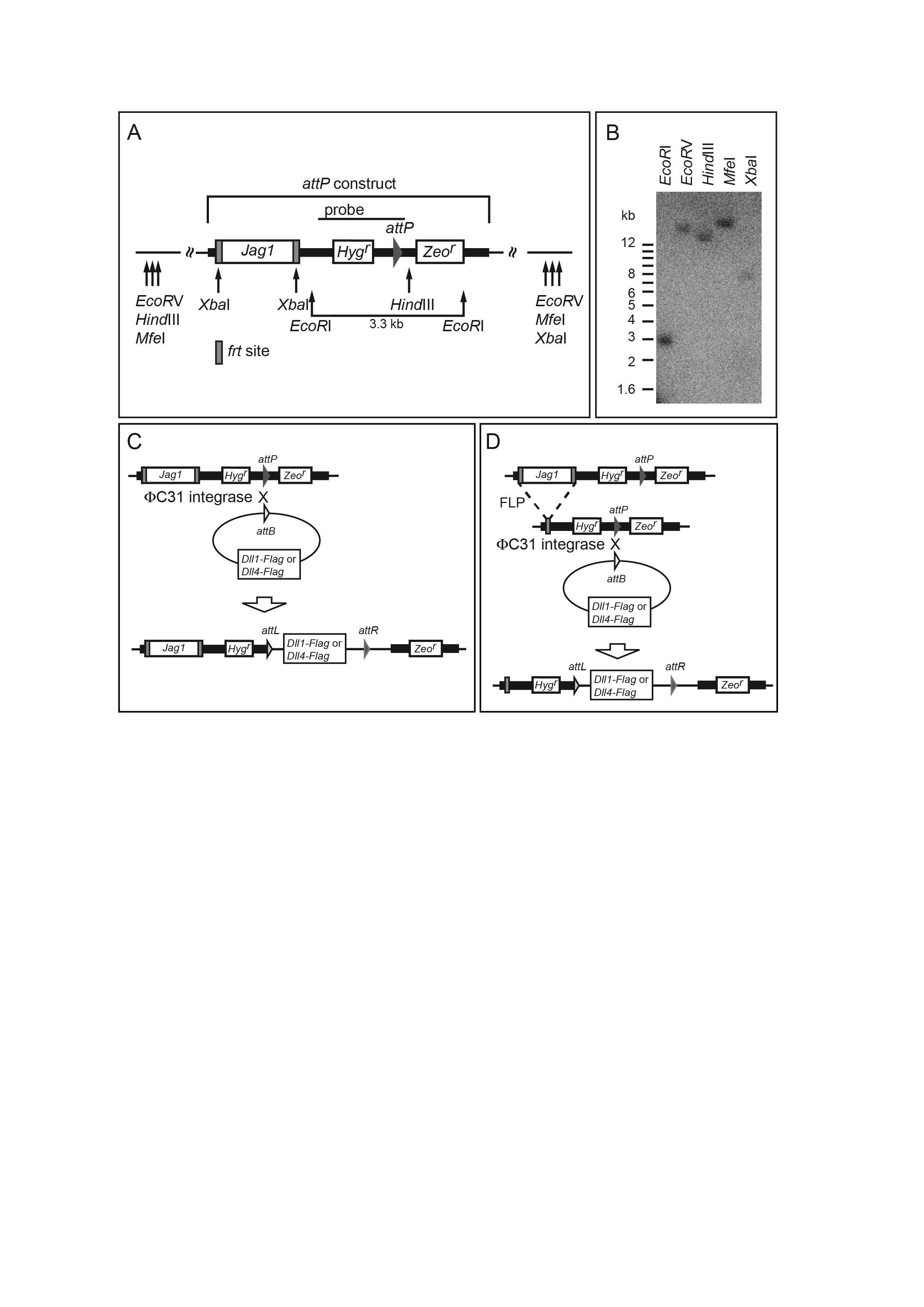

Supplement: S4 Fig — (A) Map of the genomic integration of the pHZ-attP construct containing attP site, Hyg r, Zeo r and frt-flanked Jagged1 (Jag1). The position of restriction sites and of the probe used for Southern blot analysis are indicated. The flanking genomic sequence and position of restriction sites outside the vector is unknown. (B) Southern blot analysis of DNA isolated from CHOattP-JAG1 cells shows a single product for each digest indicating a single genomic integration of the attP construct (the 3.3 kb EcoRI fragment is entirely derived from the integrated construct and served as a control). (C) ΦC31 integrase-mediated insertion of Dll1 and Dll4 into CHOattP-JAG1 generates CHOattP-JAG1-DLL1 or CHOattP-JAG1-DLL4 cells used in S6D Fig; JAG1 is Myc-tagged, DLL1 and DLL4 are Flag-tagged. (D) Excision of Jag1 by FLP recombination results in CHOattP cells that were subsequently used for the generation of CHOattP-DLL1 and CHOattP-DLL4 cells. (TIF) [file pgen.1005328.s004.tif]

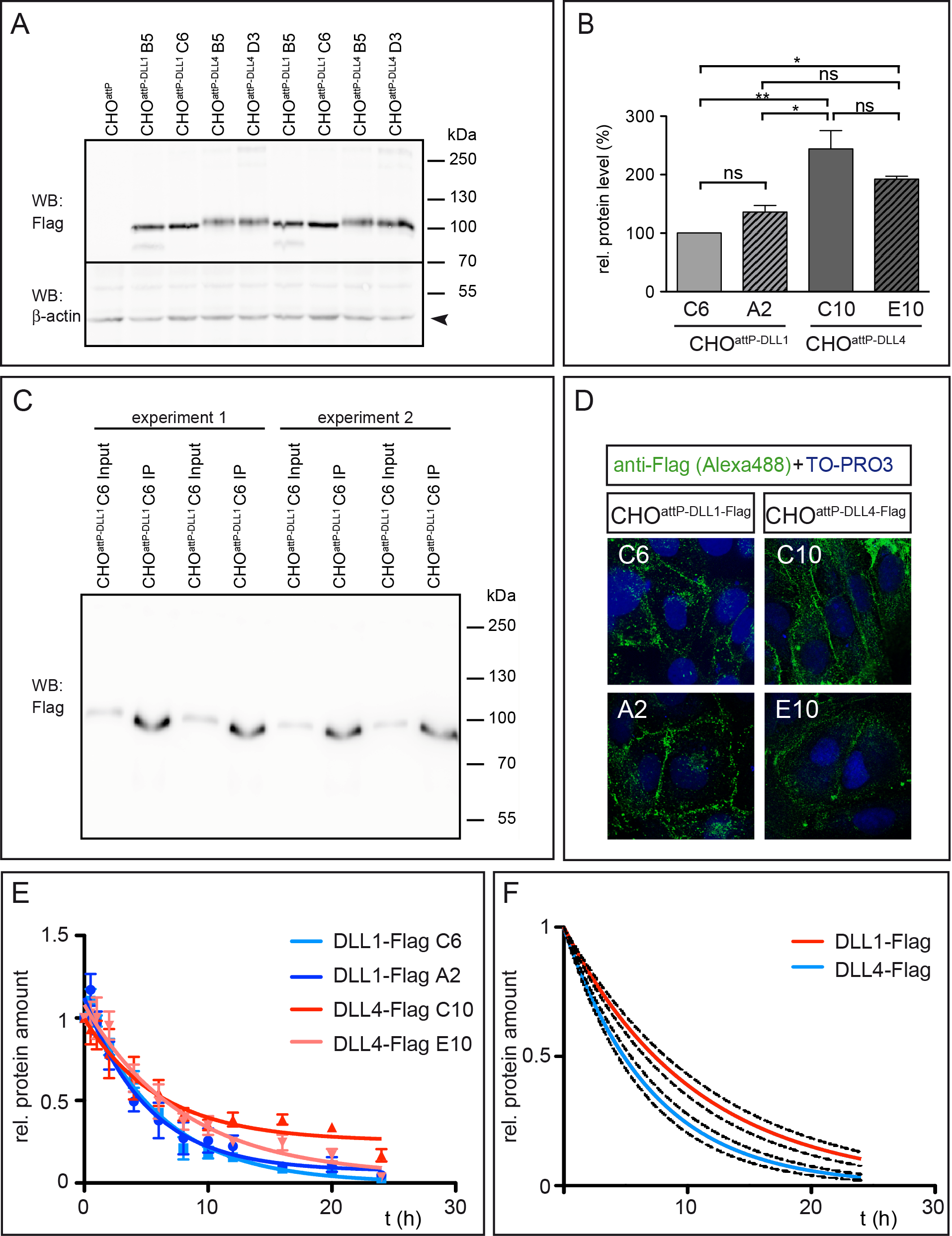

Supplement: S5 Fig — (A) Exemplary Western blot used for the analysis of protein levels in Fig 5B. CHOattP cells were used as negative control; β-actin was used for normalisation. (B) Extended Western blot analysis of protein levels including additional clones of CHOattP-DLL1 and CHOattP-DLL4. Expression levels varied to some degree, but DLL4 levels were not below DLL1 levels. Clone CHOattP-DLL1 C6 is the same in Fig 5B and can be used to compare values between both Figs. Error bars represent SEM; ns, not significant; *, P<0.05; **, P<0.01. (C) Exemplary Western blot used for the quantification of cell surface protein levels by biotinylation in Fig 5C. The protein amount was quantitated and the relative protein surface level was calculated as described in Materials and Methods. (D) Immunocytochemistry of fixed CHOattP-DLL1 and CHOattP-DLL4 cells. Flag-tagged ligands were visualised using anti-Flag antibodies. DLL1 and DLL4 are present at the cell surface. (E,F) Determination of DLL1 and DLL4 protein half-lives. (E) DLL1 and DLL4 half-lives analysed using two different clones for each cell line. (F) Average protein decay of the clones shown in (E): DLL4 is more stable (half-life 7.3 hours) than DLL1 (half-life 4.9 hours). Dashed lines indicate the 95% confidence interval. (TIF) [file pgen.1005328.s005.tif]

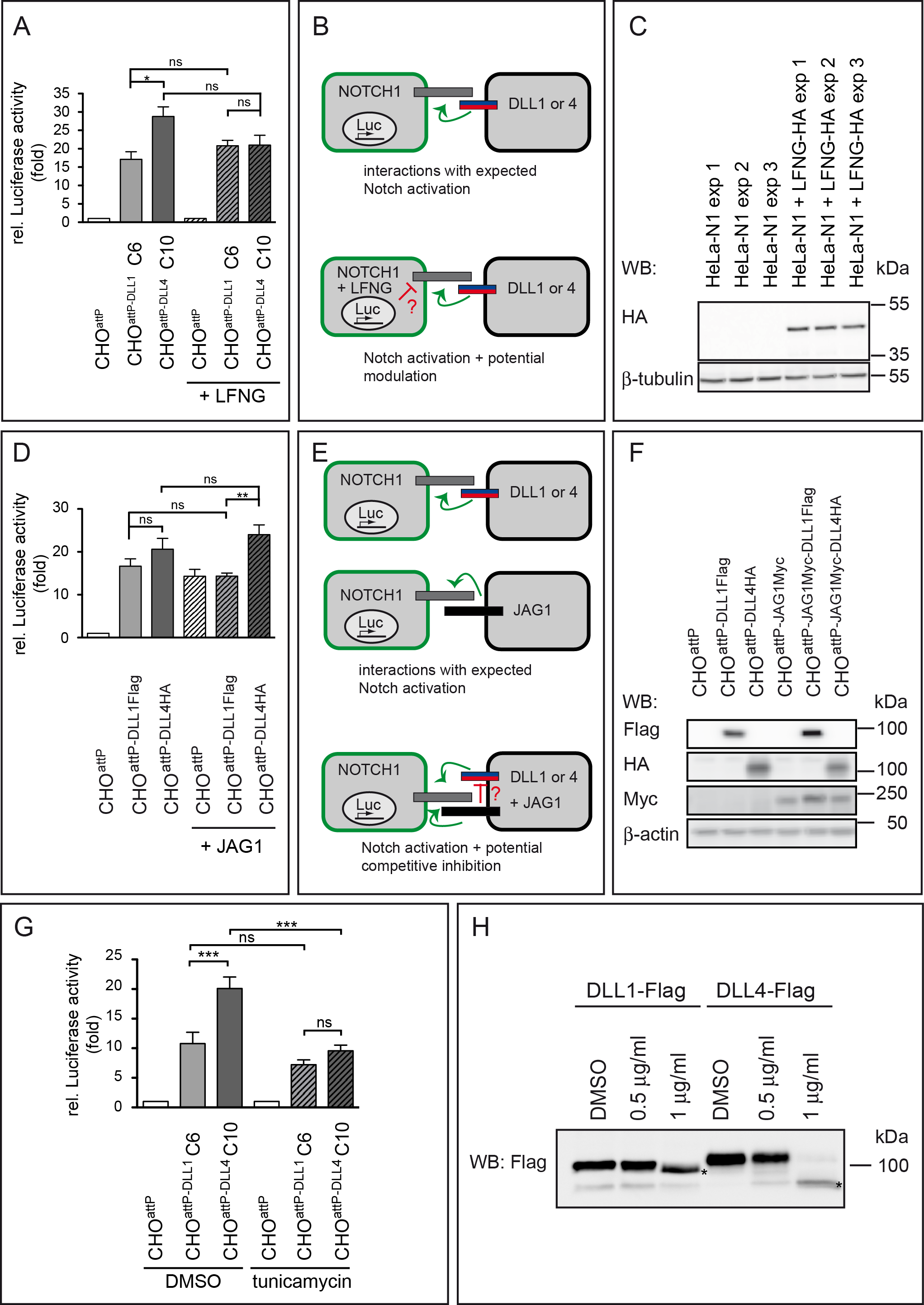

Supplement: S6 Fig — (A) Notch trans-activation assays with co-cultures of CHOattP (negative control), CHOattP-DLL1 and CHOattP-DLL4 cells with Notch reporter expressing HeLa-N1 cells (cf. Fig 5D) without and with transient expression of LFNG-HA. Expression of LFNG in HeLa-N1 cells decreases the trans-activation ability of DLL4 to levels similar to DLL1, whose activation potential is slightly increased. (B) Scheme of interactions in co-cultivation assays with possible influence of LFNG expressed in HeLa-N1 cells. (C) Western blot showing the expression of LFNG-HA in HeLa-N1 cells used in (A); β-tubulin, loading control. (D) Notch trans-activation assays with CHOattP, CHOattP-DLL1-Flag and CHOattP-DLL4-HA cells without or with stable expression of JAG1-Myc (S4C and S4D Fig) in co-culture with Notch reporter expressing HeLa-N1 cells. Stable coexpression of JAG1 in DLL1 or DLL4 presenting cells does not significantly change Notch activation. DLL4 plus JAG1 activate the receptor more efficiently than DLL1 plus JAG1. (E) Scheme of different possible interactions in co-cultivation assays with or without stable JAG1 expression. (F) Western blot showing the expression of DLL1-Flag, DLL4-HA and JAG1-Myc used in (D). β-actin, loading control. (G) Notch trans-activation assay with co-cultures of CHOattP, CHOattP-DLL1-Flag and CHOattP-DLL4-Flag cells with Notch reporter expressing HeLa-N1 cells in the absence or presence of tunicamycin, an inhibitor of N-linked glycosylation. Cultivation in the presence of 1 μg/ml tunicamycin (dissolved in DMSO) decreases the activating potential of both DLL1 and DLL4 to similar levels. (H) Treatment with 1 μg/ml tunicamycin results in a size shift of DLL1 and DLL4 (asterisks) indicating an efficient block of N-glycosylation. DLL1 and DLL4 contain one and five N-glycosylation sites, respectively; consistently, the shift is stronger for DLL4. Error bars represent SEM; ns, not significant; *, P<0.05; **, P<0.01, ***,P<0.001. (TIF) [file pgen.1005328.s006.tif]

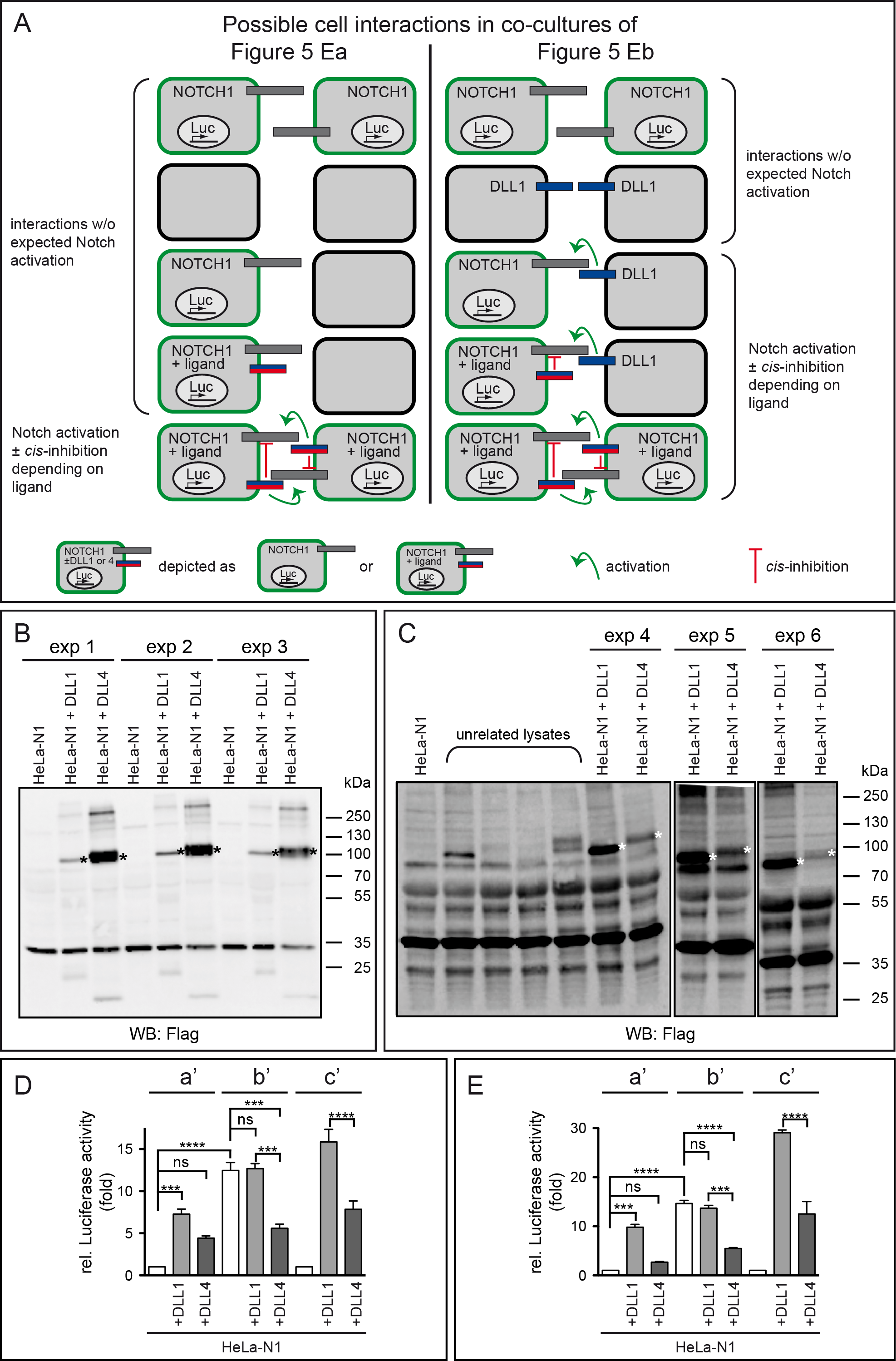

Supplement: S7 Fig — (A) Schema of all different cell interactions possible in co-cultivation assays with untransfected and transfected HeLa-N1 cells, CHOattP cells and CHOattP-DLL1 cells shown in Fig 5Ea (left) and 5Eb (right). (B,C) Transient expression of ligands was tested by Western blot analysis with anti-Flag antibodies (DLL1 and DLL4 labelled with asterisks). As expected, transient expression levels vary; in some experiments (exp 1–3; B), DLL4 was expressed more strongly than DLL1, while in other experiments (exp 4–6; C) DLL1 was expressed more strongly than DLL4. Cells of all six transfections shown were used in Notch activation assays shown in Fig 5E (n = 6). Separate analyses of Notch activation assays performed with cells that express either (D) DLL4 (B; n = 3) or (E) DLL1 (C; n = 3) more strongly result in diagrams very similar to Fig 5E, demonstrating that Notch activation assays were robust and independent of transient expression levels; a’,b’,c’ in (D,E) refer to a,b,c in Fig 5E. Error bars represent SEM; ns, not significant; *, P<0.05; **, P<0.01; ***, P<0.001; ****, P<0.0001. (TIF) [file pgen.1005328.s007.tif]

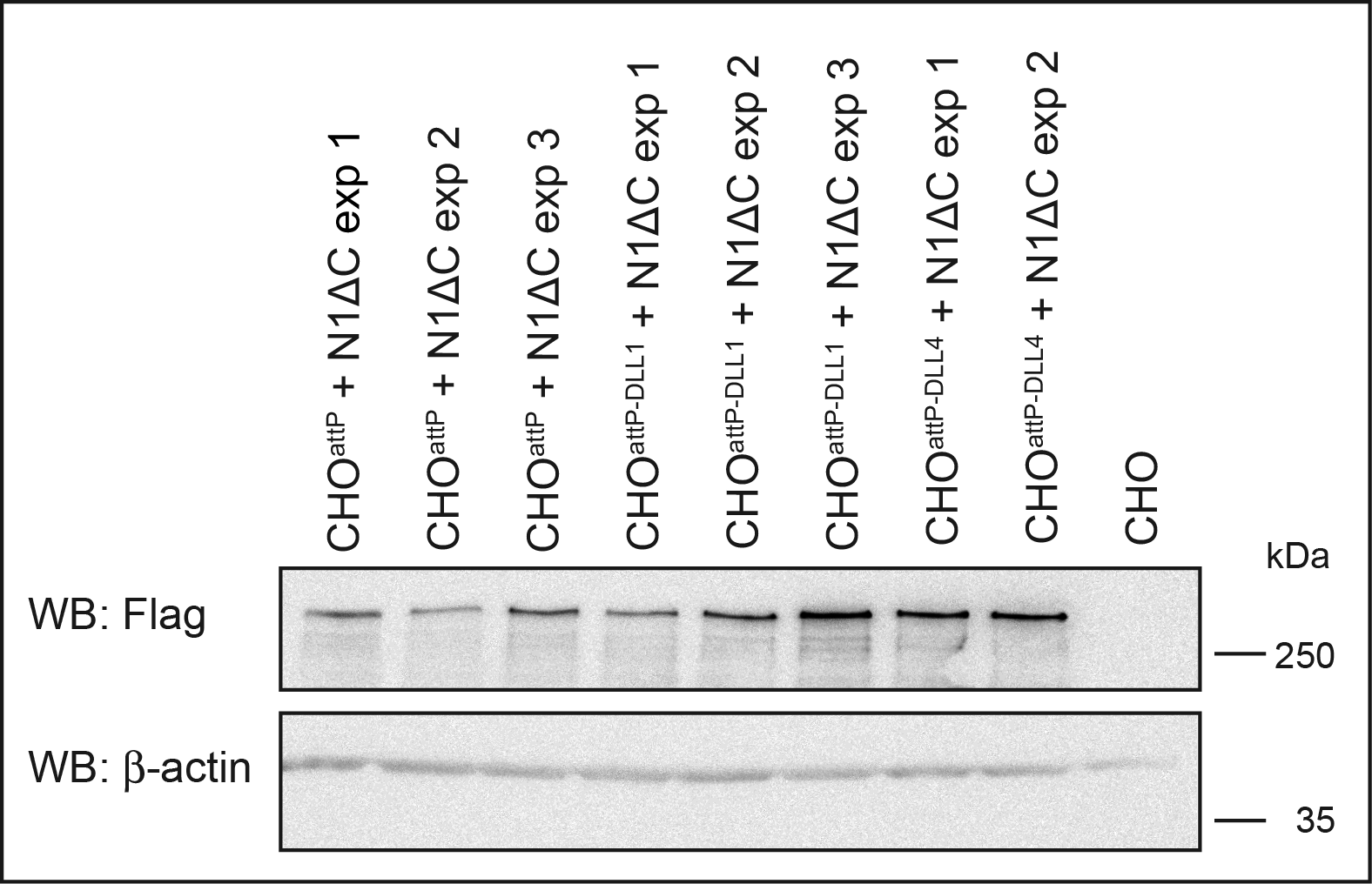

Supplement: S8 Fig — Lysates of CHOattP-DLL1 or CHOattP-DLL4 cells transiently transfected with Notch1-Flag (N1∆C) were analysed on a Western blot with anti-Flag antibodies. All transfected cell populations (used in Fig 5F) express NOTCH1. CHO, negative control; β-actin, loading control. (TIF) [file pgen.1005328.s008.tif]

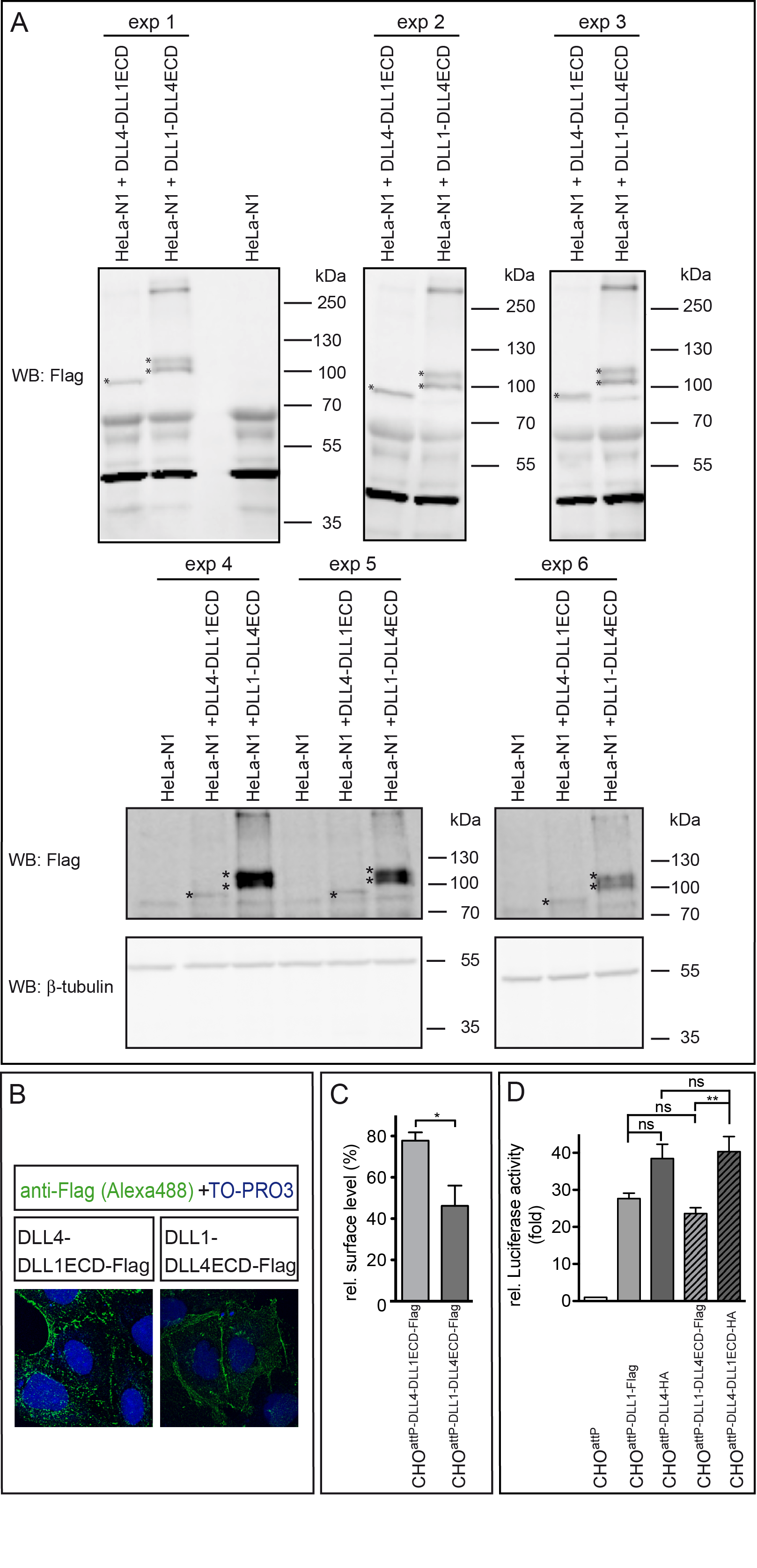

Supplement: S9 Fig — (A) Expression of chimeric ligands in transiently transfected HeLa-N1 cells was checked by Western blot analysis with anti-Flag antibodies. Lysates of cells from all transfections (exp 1–6) used in Fig 5G are shown, DLL4-DLL1ECD and DLL4-DLL1ECD signals indicated with asterisks; HeLa-N1, untransfected negative control; Flag background signal or β-tubulin, loading control. (B) Immunofluorescence of CHOattP cells stably expressing Flag-tagged chimeric ligands with anti-Flag antibodies (Alexa488-conjugated secondary antibody, green; nuclei stained with TO-PRO3, blue) shows cell surface localisation of both. (C) Surface biotinylation assays of chimeric proteins stably expressed in CHOattP cells indicate higher cell surface levels of DLL4-DLL1ECD than of DLL1-DLL4ECD; *, P<0.05. (D) Notch trans-activation assays with CHOattP cells, cells stably expressing non-chimeric proteins (CHOattP-DLL1-Flag, CHOattP-DLL4-HA) and cells expressing chimeric proteins (CHOattP-DLL1-DLL4ECD-Flag and CHOattP-DLL4-DLL1ECD-HA cells) co-cultured with Notch-reporter containing HeLa-N1 cells. The results show that all ligands, non-chimeric or chimeric, activate Notch; DLL4 and DLL4-DLL1ECD are slightly more efficient activators in this assay. Error bars represent SEM; ns, not significant; **, P<0.01. (TIF) [file pgen.1005328.s009.tif]
