# Supplementary material for: Context-Dependent Functional Divergence of the Notch Ligands DLL1 and DLL4 In Vivo
Source: PLoS Genet. 2015 Jun 26;11(6):e1005328. doi: 10.1371/journal.pgen.1005328 (PMC4482573; doi:10.1371/journal.pgen.1005328)
Supplement: S1 Table — In two independent experiments six E8.5 embryos of each genotype were lysed in 2x sample buffer and analysed by Western blot using anti-GFP and anti-β-actin antibodies. The signals were quantified using ImageJ software. The GFP signals were divided by β-actin for normalisation of different amounts loaded; additionally, the values of each experiments were divided by the CAG:Dll1 value for normalisation. (PDF) [file pgen.1005328.s010.pdf]

S1 Table. Raw data of GFP protein level analysis in Fig. 1C.

**Experiment 1**	**GFP**	**β-actin**	**GFP/β-actin**	**Normalised to *CAG:Dll1***
CAG:Dll1	11845782	19858380	0.5965	1
CAG:Dll4	2914104	6846581	0.4256	0.7135
**Experiment 2**	**GFP**	**β-actin**	**GFP/β-actin**	**Normalised to *CAG:Dll1***
CAG:Dll1	15624823	12636874	1.2364	1
CAG:Dll4	12975966	15707430	0.8261	0.6681
