# Supplementary material for: Context-Dependent Functional Divergence of the Notch Ligands DLL1 and DLL4 In Vivo
Source: PLoS Genet. 2015 Jun 26;11(6):e1005328. doi: 10.1371/journal.pgen.1005328 (PMC4482573; doi:10.1371/journal.pgen.1005328)
Supplement: S2 Table — In three independent experiments, confluent embryonic stem cells on 6 cm dishes were lysed in sample buffer and analysed on Western blots using anti-HA- and anti-β-tubulin antibodies. Three embryonic stem cell clones expressing DLL1-HA and DLL4-HA from the recombined Hprt locus were analysed; every lysate was loaded twice (#WB). HA and β-tubulin signals were quantified using ImageJ software. HA signals were divided by β-tubulin signals (normalisation of different amounts loaded) and the average value of every clone in every experiment was calculated and analysed using Prism software (GraphPad). (PDF) [file pgen.1005328.s011.pdf]

S2 Table. Raw data of DLL1-HA and DLL4-HA protein level analysis in Fig. 1D.

Experiment 1	#WB	HA	β-tubulin	HA/β-tubulin	Average
DLL1-HA A11	1	11313.309	13571.338	0.8336	0.7871
	2	8341.53	11263.51	0.7406	
DLL1-HA B2	1	14646.915	9829.731	1.4901	1.3643
	2	12850.89	10375.85	1.2385	
DLL1-HA B7	1	17604.693	9987.267	1.7627	1.5611
	2	17869.87	13145.34	1.3594	
DLL4-HA A1	1	15428.986	10717.146	1.4397	1.2859
	2	15321.92	13533.39	1.1322	
DLL4-HA A10	1	6066.510	12052.095	0.5034	0.4283
	2	4740.05	13414.95	0.3533	
DLL4-HA B10	1	11478.158	10801.610	1.0626	1.0298
	2	11298.55	11333.95	0.9969	
**Experiment 2**	**#WB**	**HA**	**β-tubulin**	**HA/β-tubulin**	**Average**
DLL1-HA A11	1	12564.501	15835.551	0.7934	0.8167
	2	13869.04	16511.48	0.8400	
DLL1-HA B2	1	16023.037	12015.510	1.3335	1.5388
	2	21009.77	12046.51	1.7441	
DLL1-HA B7	1	19099.936	13235.823	1.4430	1.6154
	2	22885.86	12800.95	1.7878	
DLL4-HA A1	1	15270.643	16397.894	0.9313	1.3652
	2	26767.34	14877.36	1.7992	
DLL4-HA A10	1	5595.933	16649.794	0.3361	0.4014
	2	7015.20	15034.12	0.4666	
DLL4-HA B10	1	3909.790	14045.480	0.2784	0.3449
	2	5888.98	14316.72	0.4113	
**Experiment 3**	**#WB**	**HA**	**β-tubulin**	**HA/β-tubulin**	**Average**
DLL1-HA A11	1	13836.480	14049.217	0.9849	0.9416
	2	11410.57	12701.19	0.8984	
DLL1-HA B2	1	14391.359	11890.560	1.2103	1.2300
	2	17071.82	13659.84	1.2498	
DLL1-HA B7	1	13343.288	11748.853	1.1357	1.1774
	2	16392.09	13445.27	1.2192	
DLL4-HA A1	1	24965.028	11123.317	2.2444	1.7986
	2	16952.11	12531.27	1.3528	
DLL4-HA A10	1	10023.137	12976.853	0.7724	0.5746
	2	4973.64	13200.56	0.3768	
DLL4-HA B10	1	11011.744	10609.974	1.0379	0.7041
	2	4680.45	12635.97	0.3704	
