# Supplementary material for: Context-Dependent Functional Divergence of the Notch Ligands DLL1 and DLL4 In Vivo
Source: PLoS Genet. 2015 Jun 26;11(6):e1005328. doi: 10.1371/journal.pgen.1005328 (PMC4482573; doi:10.1371/journal.pgen.1005328)
Supplement: S3 Table — Cells were plated on 6 cm dishes, cultured over night, lysed and analysed on Western blots with anti-Flag and anti-β-tubulin antibodies in four independent experiments. Each cell lysate was loaded twice (#WB). Two clones of each cell line were tested. Flag and β-actin signal intensities were determined using ImageJ. For normalisation, Flag signals were divided by ß-actin signals and the average for every clone in every experiment was calculated. Finally, all samples were normalized to clone CHOattP-DLL1 B5 to account for differences between the individual experiments. (PDF) [file pgen.1005328.s012.pdf]

S3 Table. Raw data of DLL1-Flag and DLL4-Flag protein level analysis in Fig. 5B.

Experiment 1	#WB	Flag	β-actin	Flag/β-actin	Average	Normalised
$CHO^{attP-DLL1}$ B5	1	11321116	4811054	2.3531	2.2898	1
	2	10426995	4683347	2.2264		
$CHO^{attP-DLL1}$ C6	1	18765480	11678832	1.6068	1.5731	0.6870
	2	20095137	13054267	1.5394		
$CHO^{attP-DLL4}$ B5	1	14444894	11596024	1.2457	1.2025	0.5252
	2	16803208	14493731	1.1593		
$CHO^{attP-DLL4}$ D3	1	15672380	7981832	1.9635	1.7217	0.7519
	2	22971179	15522459	1.4799		
**Experiment 2**	**#WB**	**Flag**	**β-actin**	**Flag/β-actin**	**Average**	**Normalised**
$CHO^{attP-DLL1}$ B5	1	9878782	9848296	1.0031	0.9322	1
	2	8276196	9607882	0.8614		
$CHO^{attP-DLL1}$ C6	1	18765480	11678832	1.6068	1.2744	1.3670
	2	10986903	11663539	0.9420		
$CHO^{attP-DLL4}$ B5	1	14444894	11596024	1.2457	1.0749	1.1531
	2	8777510	9707539	0.9042		
$CHO^{attP-DLL4}$ D3	1	10020924	7394589	1.3552	1.2531	1.3441
	2	9640631	8376004	1.1510		
**Experiment 3**	**#WB**	**Flag**	**β-actin**	**Flag/β-actin**	**Average**	**Normalised**
$CHO^{attP-DLL1}$ B5	1	8202125	6119347	1.3404	1.2163	1
	2	9348489	8558539	1.0923		
$CHO^{attP-DLL1}$ C6	1	9519004	10630832	0.8954	0.8464	0.6958
	2	11114660	13939681	0.7973		
$CHO^{attP-DLL4}$ B5	1	7794681	10045468	0.7759	0.8291	0.6816
	2	8448974	9576832	0.8822		
$CHO^{attP-DLL4}$ D3	1	8877803	8517246	1.0423	1.3111	1.0779
	2	11638874	7367004	1.5799		
**Experiment 4**	**#WB**	**Flag**	**β-actin**	**Flag/β-actin**	**Average**	**Normalised**
$CHO^{attP-DLL1}$ B5	1	10848489	5768418	1.8807	1.5485	1
	2	9980489	8205882	1.2163		
$CHO^{attP-DLL1}$ C6	1	13444782	9659246	1.3919	1.1117	0.7179
	2	11745610	14127489	0.8314		
$CHO^{attP-DLL4}$ B5	1	6149853	7042832	0.8732	0.7492	0.4838
	2	5346024	8550953	0.6252		
$CHO^{attP-DLL4}$ D3	1	11567652	6718004	1.7219	1.2618	0.8149
	2	7233045	9021439	0.8018		
