# Supplementary material for: Context-Dependent Functional Divergence of the Notch Ligands DLL1 and DLL4 In Vivo
Source: PLoS Genet. 2015 Jun 26;11(6):e1005328. doi: 10.1371/journal.pgen.1005328 (PMC4482573; doi:10.1371/journal.pgen.1005328)
Supplement: S4 Table — Protein levels of additional CHOattP-DLL1 and CHOattP-DLL4 clones were analysed as described in S3 Table. (PDF) [file pgen.1005328.s013.pdf]

S4 Table. Raw data of DLL1-Flag and DLL4-Flag protein level analysis in S5B Fig.

Experiment 1	#WB	Flag	β-tubulin	Flag/β-tubulin	Average	Normalised
$CHO^{attP\text{-}DLL1}$ C6	1	10608368	10269832	1.0330	1.0499	1
	2	10414338	9761518	1.0669		
$CHO^{attP\text{-}DLL1}$ A2	1	10632489	9578004	1.1101	1.1922	1.1355
	2	10277853	8065518	1.2743		
$CHO^{attP\text{-}DLL4}$ C10	1	14040288	7663225	1.8322	1.9105	1.8197
	2	18335087	9218690	1.9889		
$CHO^{attP\text{-}DLL4}$ E10	1	16350066	7611933	2.1480	2.1273	2.0261
	2	20154986	9567518	2.1066		
**Experiment 2**	**#WB**	**Flag**	**β-tubulin**	**Flag/β-tubulin**	**Average**	**Normalised**
$CHO^{attP\text{-}DLL1}$ C6	1	8626368	11789125	0.7317	0.7114	1
	2	7205660	10426175	0.6911		
$CHO^{attP\text{-}DLL1}$ A2	1	10490196	9001004	1.1654	1.0380	1.4590
	2	8889246	9763104	0.9105		
$CHO^{attP\text{-}DLL4}$ C10	1	17101187	8587326	1.9914	1.9080	2.6820
	2	16910380	9268054	1.8246		
$CHO^{attP\text{-}DLL4}$ E10	1	13892066	9399054	1.4780	1.3335	1.8744
	2	12283894	10331882	1.1889		
**Experiment 3**	**#WB**	**Flag**	**β-tubulin**	**Flag/β-tubulin**	**Average**	**Normalised**
$CHO^{attP\text{-}DLL1}$ C6	1	4912397	8657054	0.5674	0.7825	1
	2	5581569	5594962	0.9976		
$CHO^{attP\text{-}DLL1}$ A2	1	7178175	9908953	0.7244	1.1633	1.4865
	2	8111175	5062841	1.6021		
$CHO^{attP\text{-}DLL4}$ C10	1	13640146	7107811	1.9190	2.2054	2.8183
	2	14883388	5973154	2.4917		
$CHO^{attP\text{-}DLL4}$ E10	1	11103024	6506962	1.7063	1.4612	1.8673
	2	10403681	8554518	1.2162		
