# Supplementary material for: Context-Dependent Functional Divergence of the Notch Ligands DLL1 and DLL4 In Vivo
Source: PLoS Genet. 2015 Jun 26;11(6):e1005328. doi: 10.1371/journal.pgen.1005328 (PMC4482573; doi:10.1371/journal.pgen.1005328)
Supplement: S5 Table — Lysates from biotinylation assays were analysed on Western blots and signals were quantified using ImageJ software. Each lysate was analysed at least twice (#WB) and the average was used as result from the respective experiment. (PDF) [file pgen.1005328.s014.pdf]

S5 Table. Raw data of DLL1-Flag and DLL4-Flag cell surface level analysis in Fig. 5C.

Experiment 1	#WB	Input	IP	IP/Input	Average
$CHO^{attP-DLL1}$ B5	1	92279	49462	0.5360	0.2754
	2	387527610	44679030	0.1153	
	3	173913650	30400540	0.1748	
$CHO^{attP-DLL1}$ C6	1	108429	41992	0.3873	0.5353
	2	148838	111443	0.7488	
	3	171242	97006	0.5665	
	4	73121310	37619120	0.5145	
	5	251942280	115826400	0.4597	
$CHO^{attP-DLL4}$ B5	1	50354	30846	0.6126	0.3808
	2	37735330	6635770	0.1759	
	3	17359160	6146980	0.3541	
$CHO^{attP-DLL4}$ D3	1	146701470	82655690	0.5634	0.5748
	2	266405460	107848320	0.4048	
	3	78900	56736	0.7191	
	4	74117	45364	0.6120	
**Experiment 2**	**#WB**	**Input**	**IP**	**IP/Input**	**Average**
$CHO^{attP-DLL1}$ B5	1	46936	16890	0.3599	0.5446
	2	1095813410	198380070	0.1810	
	3	1082369390	177990570	0.1644	
	4	21819150	32141275	1.4731	
$CHO^{attP-DLL1}$ C6	1	171661	60025	0.3497	0.6023
	2	93753	37276	0.3976	
	3	95944	91237	0.9509	
	4	153723	93268	0.6067	
	5	159018790	32847900	0.2066	
	6	87843650	96852960	1.1026	
$CHO^{attP-DLL4}$ B5	1	146701470	82655690	0.5634	0.3874
	2	510376776	107848320	0.2113	
$CHO^{attP-DLL4}$ D3	1	131386	106632	0.8116	0.4282
	2	132002	48989	0.3711	
	3	37735330	6635770	0.1759	
	4	17359160	6146980	0.3541	
**Experiment 3**	**#WB**	**Input**	**IP**	**IP/Input**	**Average**
$CHO^{attP-DLL1}$ B5	1	64066	55288	0.8630	0.5037
	2	284265460	86351750	0.3038	
	3	241246610	83080540	0.3444	
$CHO^{attP-DLL1}$ C6	1	408055970	66970830	0.1641	0.1139
	2	471622465	30068610	0.0638	
$CHO^{attP-DLL4}$ B5	1	148822060	46141250	0.3100	0.3439
	2	117896140	44540040	0.3778	
$CHO^{attP-DLL4}$ D3	1	255492715	133656310	0.5231	0.4952
	2	279321375	130528030	0.4673	
**Experiment 4**	**#WB**	**Input**	**IP**	**IP/Input**	**Average**
$CHO^{attP-DLL1}$ B5	1	70740	53211	0.7522	0.5349
	2	237613620	99708820	0.4196	
	3	198719290	86037820	0.4330	
$CHO^{attP-DLL4}$ B5	1	2001344	281591	0.1407	0.1126
	2	2268016	191738	0.0845	
