# Supplementary material for: Context-Dependent Functional Divergence of the Notch Ligands DLL1 and DLL4 In Vivo
Source: PLoS Genet. 2015 Jun 26;11(6):e1005328. doi: 10.1371/journal.pgen.1005328 (PMC4482573; doi:10.1371/journal.pgen.1005328)
Supplement: S6 Table — Biotinylation assays were performed to determine the relative cell surface levels of the chimeric proteins. The lysates were analysed on Western blots twice (#WB) and the average was calculated. (PDF) [file pgen.1005328.s015.pdf]

S6 Table. Raw data of cell surface level analysis of chimeric DLL1-DLL4 proteins in S8C Fig.

Experiment 1	#WB	Input	IP	IP/Input	Average
$CHO^{attP\text{-}DLL4\text{-}DLL1ECD}$ A3	1	103365890	102099530	0.9877	0.8768
	2	101533150	77762250	0.7659	
$CHO^{attP\text{-}DLL1\text{-}DLL4ECD}$ A13	1	382498120	135309240	0.3538	0.3039
	2	322815130	82001460	0.2540	
**Experiment 2**	**#WB**	**Input**	**IP**	**IP/Input**	**Average**
$CHO^{attP\text{-}DLL4\text{-}DLL1ECD}$ A3	1	105740890	97323680	0.9204	0.7746
	2	114548150	72024680	0.6288	
$CHO^{attP\text{-}DLL1\text{-}DLL4ECD}$ A13	1	251150550	158620660	0.6316	0.6206
	2	325704460	198534510	0.6096	
**Experiment 3**	**#WB**	**Input**	**IP**	**IP/Input**	**Average**
$CHO^{attP\text{-}DLL4\text{-}DLL1ECD}$ A3	1	152163305	106294180	0.6986	0.7185
	2	121997480	90078820	0.7384	
$CHO^{attP\text{-}DLL1\text{-}DLL4ECD}$ A13	1	313683635	146456310	0.4669	0.4370
	2	251273385	102285600	0.4071	
